# Low-Dimensional Ionotronics: Focus Review

**DOI:** 10.1021/acs.chemrev.6c00092

**Published:** 2026-09-03

**Authors:** Svyatoslav Kondrat, Yuan Zhang, Yury Gogotsi, Alexei A. Kornyshev

**Affiliations:** † Institute of Physical Chemistry, Polish Academy of Sciences, 01-224 Warsaw, Poland; ‡ Institute for Computational Physics, University of Stuttgart, 70569 Stuttgart, Germany; ¶ A.J. Drexel Nanomaterials Institute, Drexel University, Philadelphia, Pennsylvania 19104, United States; § Department of Chemistry, Molecular Sciences Research Hub, White City Campus, London W12 0BZ,United Kingdom

## Abstract

Ionotronics encompasses a broad range of phenomena and devices based either on the electron-controlled manipulation of ions in electrolytic media or on the use of ionic behavior to control electronic functionality. Although the term itself is relatively recent, the scientific foundations of ionotronics span more than a century of research. Owing to their promising applications in areas ranging from energy storage and harvesting to robotic actuation, nanofiltration, water desalination, sensing, and neuromorphic computation, ionotronic systems have recently attracted rapidly growing attention, particularly in the context of low-dimensional electrodes. These advanced architectures can profoundly modify the electrode–electrolyte interface, where ion transport and interfacial phenomena play a central role. Such systems exhibit distinctive physics arising either from nanoscale confinement, which restricts the electrolyte to only a few layers of ions or solvent molecules, or from low-dimensional electronic effects imposed by confining materials such as graphene, carbon nanotubes, and MXenes. This Focus Review aims to provide a comprehensive yet accessible overview of the fundamental phenomena governing low-dimensional ionotronic systems, with particular emphasis on the underlying physics that determines their performance and enables emerging applications.

## Graphical abstract

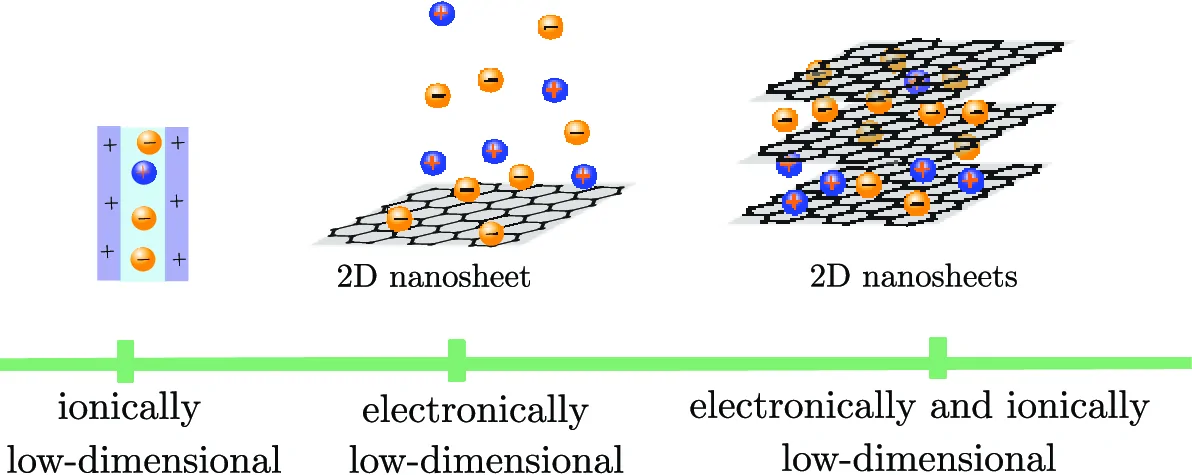

## 1 Introduction

The past four decades have ushered in the era of nanomaterials, beginning with the discovery of fullerenes,^1^ followed by carbon nanotubes,^2^ graphene,^3^ and a wide variety of inorganic and organic 0D, 1D, and 2D systems. Many of these materials can confine water or organic solvents and accommodate mobile ions within subnanometer pores and interlayer spacing, giving rise to ionotronic systems—*electronically conductive* solids hosting fluids and ions under extreme confinement. Unlike conventional intercalation compounds, such systems allow reversible migration of ions and solvent molecules under an applied potential. They also fundamentally differ from materials such as clays or biological ion channels, which likewise confine electrolytes in low-dimensional geometries but lack electronic conductivity. Furthermore, as discussed throughout this Focus Review, the behavior of low-dimensional ionotronic systems differs qualitatively from that of electrolyte-filled *disordered* microporous carbons. Ironically, however, theoretical frameworks originally developed for disordered carbon electrodes^4–9^ —while often too idealized to faithfully describe the structural complexity of such materials—prove to be much better suited for structurally well-defined low-dimensional ionotronic systems. Although these simplified approaches still cannot fully capture the chemical specificity and atomistic complexity of real nanomaterials and their interactions with ions and solvents, they nevertheless identify the key physical mechanisms governing low-dimensional ionotronics and provide a conceptual framework for understanding their behavior.

This Focus Review concentrates on systems in which mobile, typically solvated ions are confined within ordered, conductive nanostructures. While low-dimensional electrodes—such as carbon nanotubes or reduced graphene oxide—have been widely studied in various electrolytes, a unified framework for understanding their fundamental properties remains lacking. Given the rapid progress in this field, an overview of their common features and underlying principles is both timely and essential. As novel ionotronic nanomaterials advance into the fields ranging from energy storage and harvesting to separation technologies, electronics, and healthcare, it is becoming increasingly important to have a critical look at the laws and processes governing voltage-tunable ion manipulation in subnanometer confinement. With the rapid expansion of 2D materials beyond graphene and metal chalcogenides—particularly the discovery of MXenes^10,11^ —the scientific community now has access to a large variety of electronically conductive, mechanically robust, and environmentally stable 2D building blocks with highly tunable properties, offering unprecedented opportunities for designing advanced ionotronic systems. The quantum properties of low-dimensional materials combined with the extreme mechanical strength of 1D and 2D nanostructures will open the door to a wide range of applications, including ionic transistors and memristors, bioelectronic devices, electrochromic coatings, modulation of RF and IR reflectivity, tuning frequency of RF antennas, enhanced electrochemical actuators, structural batteries and supercapacitors, wearable electronics, *etc*.

Advancing low-dimensional ionotronic technologies requires a profound understanding of the quantum mechanical, dynamical, and electrochemical properties that determine their performance. In this Focus Review, we propose a classification of low-dimensional ionotronics based on their confining characteristics and provide a comprehensive yet concise overview of the electrode materials, physicochemical phenomena, and emerging applications that underpin these systems. Our goal is to present an accessible but rigorous perspective that will be valuable to researchers from diverse disciplines—including materials science, chemistry, and physics—as well as to engineers seeking to translate fundamental insights into practical technologies. By exploring the complex interplay between ionic transport, confinement, and electronic properties, we aim to establish a unifying framework for the rational design and optimization of next-generation ionotronic systems across a broad range of applications.

## 2 Types of Low-Dimensional Ionotronic Systems

To systematically discuss the behavior of low-dimensional ionotronic systems, we classify them into three distinct categories based on the dimensionality of electronic and ionic confinement (Figure 1). The first class, which we term *ionically* low-dimensional ionotronics, comprises systems in which the electrodes impose strong spatial confinement on ions while remaining electronically three-dimensional conductors. A key, yet often underappreciated, aspect of such systems is that fluids under extreme confinement can exhibit properties fundamentally different from those in the bulk. Counterintuitively, confined electrolytes may even acquire solid-like characteristics when ions and solvent molecules interact strongly with the confining surfaces.^12^ Conversely, when wall interactions are weak—as in hydrophobic or ionophobic carbon nanotubes—ions and solvent molecules may move collectively in a single-file manner.

We therefore regard confinement as low-dimensional only when it substantially modifies the properties of the confined electrolyte. As the confinement size increases, a bulk-like region gradually develops in the center of the pore, and the system approaches the behavior of an electrolyte near a flat interface (Figure 1a), which is comparatively well understood within existing theoretical and simulation frameworks. A universal geometric criterion for low-dimensional confinement is difficult to establish, as the crossover depends on ion size, solvent properties, and the specific interactions between the electrolyte and the confining material. Nevertheless, available evidence suggests that bulk-like, quasi-homogeneous regions begin to emerge once the confinement accommodates approximately three to five molecular layers. Throughout this Focus Review, we therefore adopt three–five molecular layers as a rough, purely conventional criterion for low-dimensional confinement, while emphasizing that this definition is neither rigorous nor universal.

In *electronically* low-dimensional ionotronics, the electrode does not spatially confine ions; instead, the electron wave functions within the electrode are confined along at least one spatial dimension. As a result, the electronic response to ionic behavior differs substantially from that in conventional 3D electrodes, leading to distinct electrode–electrolyte properties. Importantly, in electronically low-dimensional ionotronics, ions still experience an effectively 3D bulk environment, except in the immediate vicinity of the electrode interface. When ions are also spatially confined, the system belongs to the third category, namely *electronically and ionically* low-dimensional ionotronics. As we discuss below, such systems exhibit qualitatively different behavior from the other two classes.

We define an ionotronic system as low-dimensional only if it exhibits well-defined structural characteristics, as discussed above, over extended length scales. Accordingly, popular materials such as carbide-derived carbons (CDCs), as well as other disordered or even ordered porous materials such as metal–organic frameworks (MOFs), do not qualify as low-dimensional electrodes. Although these systems may display certain features associated with low-dimensional ionotronics, the presence of meso- and macropores, interconnected pore networks, and broad pore-size distributions tends to average out the distinctive properties of genuinely low-dimensional systems. Consequently, they are more appropriately regarded as 3D porous materials.

**Figure 1:**
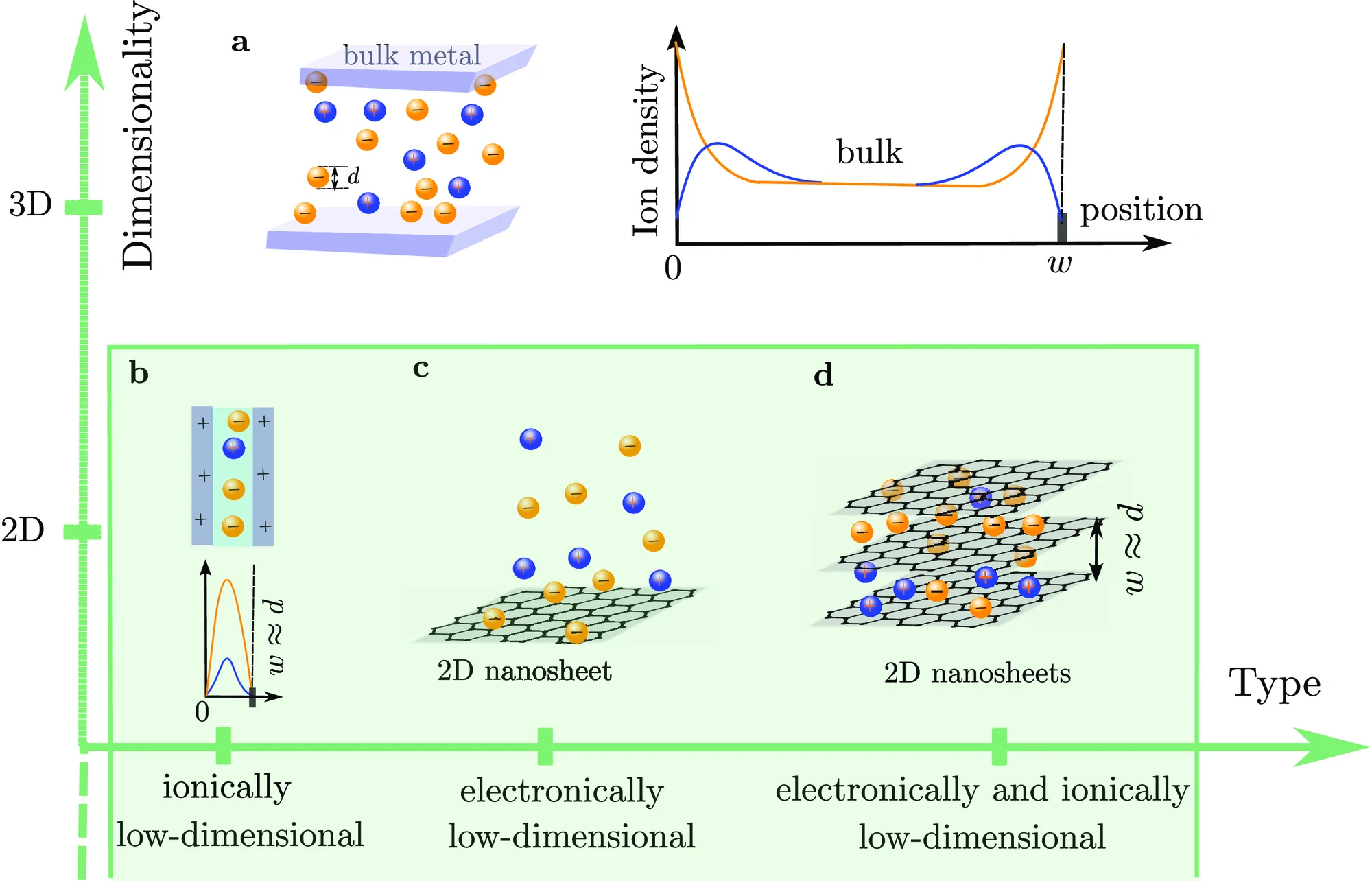
**Types of ionotronic systems.** (**a**) Conventional 3D ionotronic systems contain well-defined bulk regions. (**b–d**) Low-dimensional ionotronic systems can be classified into three categories: (**b**) ionically low-dimensional electrodes, which provide strong confinement for ions; (**c**) electronically low-dimensional electrodes, which confine electronic wave functions; (**d**) electronically *and* ionically low-dimensional electrodes, which simultaneously confine ionic and electronic degrees of freedom. This Focus Review discusses the ionotronic systems highlighted by the green box. The dashed vertical segment of the *y* axis and the open lower edge of the green box indicate the extension of this classification to 1D and 0D ionotronic systems, which are not shown explicitly (cf. Figure 3a).

## 3 Low-Dimensional Electrode Materials

Low-dimensional electrode materials possess electronic conductivity and a range of other properties that make them attractive for energy storage and conversion applications,^13^ as well as for diverse technologies spanning sensing, separations, electronics, and actuation.^14–18^ Compared to conventional 3D porous materials, they offer large accessible surface areas for ion storage together with shorter ionic diffusion pathways enabled by their highly ordered structures.^19,20^

Each class of low-dimensional materials comprises numerous nanostructural variants, including single-, double-, and multilayered architectures, oxides and reduced oxides, mixed surface terminations, *etc*. As a result, their properties can vary substantially—for example, from hydrophilic and poorly conducting to hydrophobic and highly conductive—providing broad opportunities for rational material design and tunability tailored to specific applications. Representative examples of low-dimensional electrode materials are shown in Figure 2, while the characteristics and synthesis routes specific to 0D, 1D, and 2D systems are discussed below.

### 3.1 0D Electrodes

Examples of 0D electrodes include fulerenes and onion-like carbons (OLCs). OLC are synthesized by annealing nanodiamonds at high temperatures (1200° to 2000°) in an inert atmosphere or through carbon arc discharge, resulting in concentric, multishell fullerene structures with high conductivity and surface area.^21^ These materials offer rapid ion transport, making them particularly effective for supercapacitors and fast-charging energy storage devices.^22^ However, these materials primarily provide surface-level ion confinement, with most ions located in bulk electrolyte filling the space between these electronically low-dimensional 0D particles.

Nanoporous carbon with 0D nanoconfinement can be synthesized via template-assisted methods (using silica or metal–organic frameworks), activation processes (with KOH or CO_2_), carbonization of organic precursors (e.g., polymers, biomass) at high temperatures (600° to 1000°) under inert atmospheres or selective etching of metals from various metal carbides.^23^ The porosity and surface area can be tuned by activation conditions, precursor selection, and post-treatment. The pocket-like^24^ 0D sites in these materials provide strong ionic confinement, enabling efficient energy storage.^24^ However, limited access to these sites and their tight geometrical constraints can hinder ionic transport. As 0D confining sites are parts of 3D nanoporous networks, we address them only occasionally for completeness.

### 3.2 1D Electrodes

The prime examples of 1D materials are carbon nanotubes (CNTs), distinguished by their cylindrical geometry. CNTs are mainly synthesized via chemical vapor deposition (CVD), arc discharge, or laser ablation, in which carbon-containing precursor gases (e.g., CH_4_ or C_2_H_2_) decompose at elevated temperatures (600° to 1200°) in the presence of metal catalysts such as Fe, Co, or Ni. Their structure—whether single-walled or multiwalled—is governed by the growth conditions, catalyst composition, and reaction parameters.^29^

CNTs exhibit remarkable electronic transport properties and mechanical strength, making them attractive for applications in batteries, fuel cells, and flexible energy-storage devices.^30^ In practice, however, the outer surface of CNTs is generally much more accessible than their narrow inner channels. As a result, charge storage predominantly occurs on the external surface, placing CNT electrodes in a category similar to electronically 0D particles. Although numerous theoretical and experimental studies have investigated ionic transport within CNT interiors, these inner channels typically require postsynthesis opening and therefore contribute only weakly to charge storage in conventional nanotube electrodes (cf., however, Figure 5g). Nevertheless, the inner nanotube channels can play an important role in applications such as water desalination and ion separation when incorporated into membrane architectures.

### 3.3 2D Electrodes

2D materials offer confinement of ions between nanosheets. The confinement can be dynamic with the interlayer spacing changing when ions and solvent molecules are intercalated or released. The most prominent example is graphene, a single layer of *sp* ^2^-hybridized carbon atoms arranged in a hexagonal lattice. Graphene can be made through mechanical exfoliation of graphite, CVD on metal substrates, or chemical/thermal reduction of graphene oxide, with each method balancing quality, scalability, and cost.^31^ Its extraordinarily high surface area, good electrical and thermal conductivity, and mechanical flexibility make it an outstanding candidate for conductive additives and binders in lithium-ion batteries, supercapacitors, and wearable electronics.^32^

As an elementary building block of graphite, the graphene properties have been explored theoretically^33^ long before its experimental isolation.^3^ The calculations revealed that the band structure of graphene near the Fermi level comprises valence and conduction bands, both resembling conical surfaces, which touch each other at the so-called Dirac points. Hence, graphene exhibits a zero band gap, a characteristic of semimetals or zero-gap semiconductors. Despite this, graphene demonstrates the lateral conductivity comparable to or even higher than that of many metals, owing to the high mobility of charge carriers arising from its unique band structure. In addition, graphene exhibits excellent mechanical stability and a high surface-to-volume ratio, garnering significant interest as an electrode material.^34^

A key example of 2D materials is MXenes, an emerging and rapidly growing family of 2D transition metal carbides, nitrides, and carbonitrides. MXenes are typically synthesized by selective etching of the “A” layers from MAX phases (e.g., Ti_3_AlC_2_) using HF, LiF/HCl, other acidic solutions, or molten salts, followed by delamination to obtain few-layer or single-layer 2D flakes.^35^ In addition, MXenes can be synthesized via CVD, offering an alternative route for controlled growth.^36^

**Figure 2:**
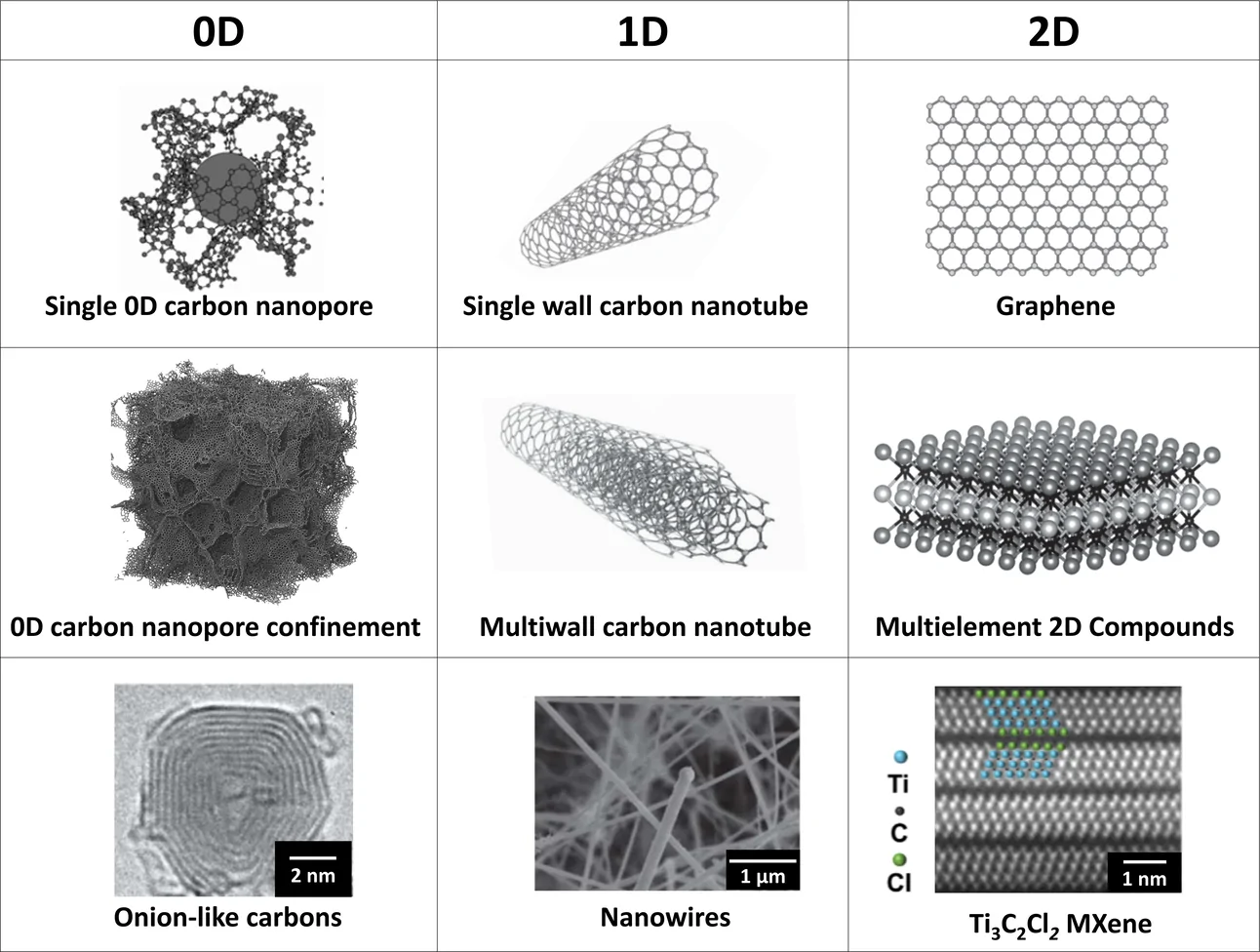
**Low-dimensional electrodes.** In 0D electrodes, ions are either trapped within a spherical cavity or adsorbed on the surface of a nanoparticle. In 1D electrodes, ions reside inside nanotubes or at the surface of nanowires. In 2D electrodes, ions are confined at the surface or within the interlayer spaces between nanosheets. The image of 0D carbon nanopores reproduced from ref 25 (available under a CC-BY-NC 4.0 license; copyright 2012 Alonso et al.) and ref 26 (available under a CC-BY 4.0 license; copyright 2017 Zhan et al.). The image of onion-like carbons reproduced with permission from ref 27. Copyright 2010 Macmillan Publishers Limited. The image of MXenes reproduced with permission from ref 28. Copyright 2023 the American Association for the Advancement of Science. All other images reproduced with permission from ref 13. Copyright 2019 the American Association for the Advancement of Science.

With their highly tunable structure and chemistry, MXenes represent a promising class of electrodes for energy storage and conversion^11,37^ and other applications.^14–18^ The combination of high metallic conductivity,^38^ redox-active transition metal-oxide-like surface, and hydrophilicity (in the case of oxygen/OH terminations) makes them truly unique among low-dimensional materials, enabling superior performance in energy-related and other ionotronic applications. As highly conductive 2D materials with a subnanometer 2D confinement and a negative surface charge, MXenes are favorable for cations insertion and can be effectively used as negative electrodes in batteries and pseudocapacitors.^39^ 2D flakes can be assembled into freestanding film electrodes with uniformly distributed slit-shape pores within the electrode, which offer a potential for fundamental studies and practical applications. The highly tunable chemistry on M (transition metals) and X (carbon and nitrogen) sites and surface terminations, T_*x*_ (=O, −OH, -Cl, -F), allow tuning their electronic structure, surface redox activity, and hydrophilicity. The subnanometer slits in 2D materials can host various intercalants due to dynamically tunable interlayer spacing.

Fluids confined within MXenes provide an essential model system to study charge storage and transport properties under confinement. For instance, the number of confined water layers can reversibly change from one to three as a function of humidity,^40^ applied pressure,^41^ and electrochemical potential.^42^ These changes are also accompanied by variations in layer spacing and the degree of ion solvation,^42^ creating a dynamically changing environment for ions. Depending on ion size and valency, MXenes can show drastically different charge storage capacities, as demonstrated experimentally and theoretically for aqueous electrolytes.^20,43^ As ions intercalate between MXene nanosheets under applied potential, the degree of ion desolvation and the distribution of ions within the confinement can influence various processes, including electrochemical charge storage,^44^ ion transport,^45^ and electron transfer.^46^ Moreover, the charge storage mechanisms depend not only on the properties of the confiner, such as pore size and surface functionality, but also on the characteristics of the confinee, including ion type, solvent type, and ionic concentration.^20,42,44,47^

Thus, 2D materials like MXenes, graphene and transition metal dichalcogenides are important electrode materials for ionotronic systems because of their unique ability to facilitate and control ionic and electronic transport in 2D nanoconfinement. These materials enable high-speed ionic responses and offer tunable surface chemistry and robust mechanical properties, essential for modern ionotronics. They also offer opportunities for computation-driven design due to their periodic structures, uniform slit pore widths between the 2D sheets, and a large variety of 2D nanosheets with diverse properties.

## 4 Ionically Low-Dimensional Systems

Ionically low-dimensional electrodes confine electrolytes to quasi-0D, 1D, or 2D geometries (Figure 3a), while retaining electronic properties—such as high electrical conductivity and quantum capacitance—comparable to those of bulk 3D electronic conductors. A particularly notable example is provided by MXenes, which can combine angstrom-scale 2D ionic confinement with exceptionally high electronic conductivity.^38^

**Figure 3:**
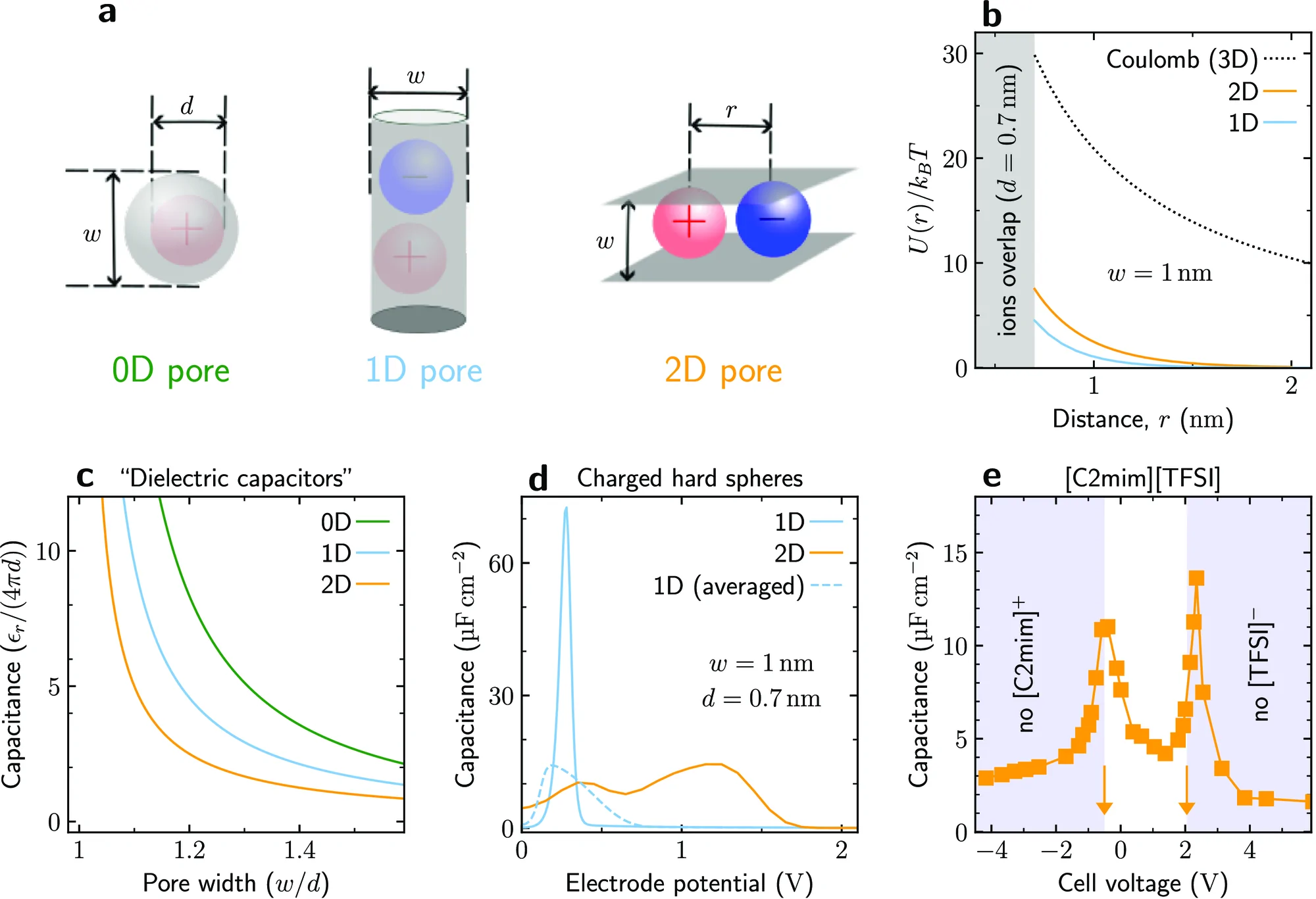
**Ionically low-dimensional ionotronics: interactions and equilibrium.** (**a**) Schematic representation of 0D, 1D, and 2D confinements. (**b**) Electrostatic interaction energy between ions in bulk and in 1D and 2D pores as a function of ion separation. (**c**) Capacitance predicted by the 0D, 1D, and 2D dielectric capacitor models, eqs 3 to 5, as a function of pore width. Capacitance is measured in units of *C*_0_ = ϵ_*r*_/(4*πd*), while the pore width *w* is expressed in units of the ion diameter *d*. (**d**) Capacitance as a function of the potential difference relative to the bulk electrolyte for 1D and 2D pores of width (diameter) *w* = 1 nm and ions of diameter *d* = 0.7 nm. Data for 2D pores are from Monte Carlo simulations.^77^ Data for 1D pores are from the analytical single-file pore model of Verkholyak.^78^ The blue dashed line shows the results for 1D pores averaged over a Gaussian pore-size distribution with a mean pore size of 1 nm and variance 0.1 nm^2^. (**e**) Capacitance as a function of cell voltage obtained from molecular dynamics (MD) simulations of [C2mim]­[TFSI] ionic liquid in an electrical double-layer capacitor with two identical electrodes comprising slit (2D) pores. The arrows indicate the voltages at which co-ions are abruptly expelled from the pores, resulting in sharp capacitance peaks. Correspondingly, the shaded areas represent voltage ranges where the pores are free of co-ions. Data from ref 8.

The well-defined periodic structures and high electronic conductivity of ionically low-dimensional materials make them particularly amenable to theoretical modeling and simulations. Periodicity enables the construction of simplified 1D or 2D models in which pores can be treated as infinitely extended, while high electronic conductivity justifies approximating pore walls as ideally metallic. These features allow for analytical treatments of ionic interactions (Section 4.1) and facilitate the use of established simulation techniques to describe the electronic response of electrode materials, such as Gaussian charges of Siepmann and Sprik^48^ and induced counter-charge (ICC) method^49^ (these approaches are implemented in several ready-to-use software packages^50–52^ ). Despite recent advances,^53–55^ comparable treatments remain scarce or largely undeveloped for more complex, electronically heterogeneous materials.

### 4.1 Interactions

Unlike ions in nonconducting confinements, such as dielectric or insulating pores, an ion confined within a low-dimensional *conducting* pore is accompanied by an induced electron cloud at the pore walls. Owing to the extreme angstrom-scale confinement, this induced electronic charge remains in the immediate vicinity of the ion, profoundly affecting electrostatic interactions, both among the confined ions and with the pore walls.

#### 4.1.1 Ion–Pore-Wall Interactions

In addition to the direct van der Waals (dispersion) ion–wall interactions, ions experience an additional attraction to conducting pore walls mediated by the conduction electrons within the confining material. To illustrate this effect, consider a single ion of charge *q* located at a distance *r* from an ideally metallic wall in the absence of confinement. The conduction electrons in the metal respond to the ion’s electric field by redistributing so as to completely screen the field inside the metal. This electrostatic response is mathematically equivalent to introducing an image charge of equal magnitude and opposite sign located at a distance *r inside* the metal. The ion and its image charge attract one another, giving rise to an effective ion–wall attraction that decays proportionally to *q* ^2^/(2*r*).

Under low-dimensional confinement, this effect becomes strongly amplified. Multiple reflections of the ionic charge and its images between the confining walls generate a series of image charges whose cumulative contribution substantially lowers the electrostatic energy of ions inside the pore. Consequently, increasing the degree of confinement progressively enhances the thermodynamic favorability of ion adsorption.

In Section 4.4.1, we further discuss how this affects pore filling and electrolyte behavior in low-dimensional pores.

#### 4.1.2 Interionic Interactions

The induced electronic charge in the immediate vicinity of the ion efficiently screens the electrostatic field generated by the ion, both across the confining walls and along the pore axis. As a result, the effective electrostatic interactions between two ions within the same pore are profoundly modified. For 1D and 2D *perfectly metallic* confinements, analytical—albeit mathematically involved—expressions for the corresponding interaction potentials are available. In the limit of large separations (*r*) between ions located near the center of the pore, these expressions simplify to^56^

1 $$\frac{U_{\alpha \gamma }^{\left(1D\right)}\left(r\right)}{k_{B}T}\approx 6.16Z_{\alpha }Z_{\gamma }\frac{l_{B}}{w}e^{-4.8r/w}$$

for 1D pores, and^4^

2 $$\frac{U_{\alpha \gamma }^{\left(2D\right)}\left(r\right)}{k_{B}T}\approx 2.83Z_{\alpha }Z_{\gamma }\frac{l_{B}}{\sqrt{rw}}e^{-\pi r/w}$$

for 2D pores. Here, α and γ denote ion species, *Z*_α_ is the valency of ion α, and *w* is the pore width (nanotube diameter). The Bjerrum length, *l*_*B*_ = *e* ^2^/(*k*_*B*_ *Tε*_r_) (in Gaussian units), defines the distance at which the electrostatic interaction between two monovalent ions equals the thermal energy *k*_*B*_ *T*, where *k*_*B*_ is the Boltzmann constant, *T* is temperature, *e* is the elementary charge, and ε_r_ is the relative permittivity of the medium emerging due to the motion of the noncharged polar part of the confined ions and solvent, if present (for effects of solvent see Section 4.4.3). It is important to note that ε_r_ depends on both pore dimensionality and pore width. Under strong confinement, the dielectric response is generally anisotropic, with distinct values parallel and perpendicular to the confining walls. Despite extensive research, no broadly accepted microscopic picture of dielectric response under strong confinement has yet emerged. This lack of consensus is reflected in reported effective dielectric permittivities spanning several orders of magnitude.^57–62^

Equations 1 and 2 show that ionic interactions decay exponentially with ion separation, reminiscent of Debye screening. However, unlike Debye screening in bulk electrolytes—which arises from rearrangements of mobile ions in the solution—the screening in low-dimensional pores is provided (to a high degree) by induced electronic charge in the pore walls, which acts as the compensating counter-charge for each ion. Interaction potentials in nonideal metals, such as those described by the Thomas-Fermi model, exhibit a similar spatial decay.^5,63,64^ Comparable behavior has also been obtained from quantum density-functional theory (DFT) calculations for cylindrical carbon and gold nanotubes, reproducing the functional form of eq 1 but with a renormalized effective nanotube radius.^65,66^

Interesting inference from eqs 1 and 2 is that screening is more pronounced in a 1D pore, i.e., in stronger confinement (the screening lengths *w*/4.8 and *w*/π for 1D and 2D pores, respectively). Figure 3b shows that this holds true at all ion separations and that both 1D and 2D pores significantly reduce interactions compared to the ‘bare’ (unscreened) Coulomb potential in 3D. This reduction of ionic interactions in low-dimensional conducting confinements, termed the *superionic state*, allows for more efficiently packing of counterions and unbinding of ion pairs and higher-order ionic complexes, thereby enhancing low-voltage capacitance for decreasing pore width and dimensionality.^4^

### 4.2 Equilibrium

We now discuss the equilibrium properties of electrolyte-filled, *ionically* low-dimensional pores, with particular emphasis on capacitance as a key characteristic for energy storage—one of the most direct and extensively studied applications of these systems.

#### 4.2.1 Capacitance

A defining feature of low-dimensional confinement is the restriction of ionic motion to one, two, or three dimensions (Figure 3a). In 1D and 2D pores, ions fluctuate within the confined directions but remain mobile along the unconstrained ones. On average, the ionic charge distribution conforms to the pore geometry and may be represented as an effective surface located at a distance *w*/2 – *a*_0_ from the pore wall, where *a*_0_ is the offset between the ion’s center of mass and its charge center. The value of *a*_0_ is typically smaller than *d*/2, where *d* is the ion diameter, and depends on the pore geometry and size.^7^ For simplicity, however, we set *a*_0_ = *d*/2, which serves the demonstrative purposes of this section. Thus, the ionic charge and the electron counter-charge form *two* parallel-plate dielectric capacitors^67^ in 2D, a cylindrical capacitor^7^ in 1D, and a spherical capacitor in 0D confinement. These textbook capacitor models provide simple analytical expressions for the capacitance. In Gaussian units, the capacitance per unit electrode surface area is given by

3 $$C_{0D}\left(w,d\right)=\frac{\varepsilon_{r}}{2\pi w\left(w/d-1\right)}$$

4 $$C_{1D}\left(w,d\right)=\frac{\varepsilon_{r}}{4\pi w\ln\left(w/d\right)}$$

and

5 $$C_{2D}=\frac{\varepsilon_{r}}{8\pi d\left(w/d-1\right)}$$

for 0D, 1D,^7^ and 2D^67^ pores, respectively. Figure 3c shows *C*_δ_ (δ ∈ {0D, 1D, 2D}) as a function of pore width *w*/*d*, demonstrating that capacitance increases with decreasing *w*, in line with the majority of experiments^68–70^ (though contrary to some other;^71–73^ the discrepancies might be due to differences in ordering of carbons^73^ or in-pore dielectric permittivity,^72^ see below). According to the dielectric capacitor model, the weakest (2D) confinement yields the lowest capacitance per surface area, and vice versa, which is consistent with the results of MD simulations showing that highly confined ions store energy more efficiently.^24^

As discussed in Section 4.1.2 (see below eqs 1 and 2), a comprehensive microscopic understanding of dielectric response under strong confinement remains lacking. While the dielectric permittivity is generally anisotropic under confinement,^57–62^ from a phenomenological perspective, increasing confinement is expected to suppress ion and solvent fluctuations while enhancing ion desolvation, thereby reducing the in-pore relative dielectric permittivity, ε_r_, below its bulk value. One may therefore expect the qualitative trend $\varepsilon_{r}^{\left(0D\right)}<\varepsilon_{r}^{\left(1D\right)}<\varepsilon_{r}^{\left(2D\right)}$ , which could in turn modify the dependence of capacitance on pore dimensionality. In addition, $\varepsilon_{r}^{\left(\delta \right)}$ is expected to decrease with decreasing pore width,^74,75^ potentially attenuating the increase of *C*_δ_ with decreasing *w* shown in Figure 3c.^72^ Such effects are particularly relevant for aqueous electrolytes, whose dielectric properties depend strongly on ion concentration and confinement, whereas they are expected to play a less pronounced role in ionic liquids. Classical density functional theory calculations support this physical picture.^76^

#### 4.2.2 Voltage-Dependent Capacitance

Like Helmholtz models of the electrical double layer, the dielectric capacitor models discussed in Section 4.2.1 (Figure 3c) neglect voltage-induced ionic rearrangements and therefore cannot capture the dependence of capacitance on electrode potential. More sophisticated approaches have been developed to account for this behavior, including analytical theories and simulations based on the electrostatic interaction potential of the superionic state, eqs 1 and 2 (see ref 79 for a review). In Figure 3d, we show the differential capacitance obtained for an electrolyte-filled 1D pore using the analytical Verkholyak model^78^ and for a 2D pore using Monte Carlo simulations,^77^ as a function of the potential difference *u* between the pore and the bulk electrolyte. The results reveal a pronounced voltage dependence of the capacitance and striking differences between the 1D and 2D cases, despite identical values of temperature, bulk chemical potentials, dielectric permittivity, and pore and ion sizes. The capacitance of a weakly polarized 1D pore nearly vanishes because entropic effects impose a free-energy penalty for ion entry into the pore. A pronounced peak emerges at *u* ≈ 0.27 V once this entropic barrier is overcome. At potentials above ca. 0.4 V, however, the capacitance rapidly decreases toward zero, reflecting near-complete saturation of the nanotube with counterions and the resulting lack of available space for further charge accumulation.

The capacitance of 2D pores exhibits even richer—though quantitatively less extreme—variations with *u*, featuring two distinct peaks before eventually vanishing upon pore saturation. Notably, saturation occurs at substantially higher potential differences than in the 1D case. This behavior is generic and indicates that 2D pores are better suited for applications requiring operation at elevated voltages.

#### 4.2.3 Phase Transition and Charging Hysteresis

A key distinction between 1D and 2D confinement is that the latter can support phase transitions in confined electrolytes, characterized by abrupt changes in ion density and capacitance.^79^ One class of such transitions, predicted by mean-field theory^4^ and validated by atomistic simulations,^8^ is illustrated in Figure 3e. As the cell voltage is swept away from zero in either direction, co-ions are abruptly expelled from the pore, resulting in pronounced capacitance peaks.

As with any first-order phase transition, co-ion desorption exhibits hysteresis, such that abrupt expulsion and adsorption of co-ions occur at different voltages during charging and discharging. Lee et al.^80^ demonstrated that such hysteretic charging leads to energy dissipation—typically a few percent per charge–discharge cycle—suggesting that these transitions are undesirable in applications where minimizing energy losses is critical. However, hysteresis can also be deliberately exploited to realize memcapacitive functionality (Section 7.10). More generally, the role of in-pore phase transitions and their associated hysteresis remains largely unexplored, representing an intriguing direction for future research.

#### 4.2.4 Low-Dimensional versus 3D Microporous Electrodes

The capacitance of 1D and 2D pores exhibits a rich variety of voltage-dependent behaviors, manifested by one or two peaks and even abrupt jumps (Figure 3d,e). Such variability has not yet been clearly identified experimentally because most studies employ amorphous, bulk-like (3D) porous carbons. Although these materials may contain 0D, 1D, and 2D micropores, their broad pore-size distributions and diverse pore geometries tend to obscure subtle features, such as phase transitions and sharp capacitance peaks.^56,81^ To illustrate this effect, the dashed line in Figure 3d shows the capacitance of 1D pores averaged over a Gaussian pore-size distribution with a mean pore width of 1 nm (corresponding to the single 1D pore shown in the same figure) and a variance of only 0.1 nm.^2^ Even such weak polydispersity leads to a much smoother voltage dependence of the capacitance. Low-dimensional electrode materials with *monodisperse* pores may therefore provide unique opportunities to isolate, investigate, and control this complex behavior, potentially enabling new practical applications.

### 4.3 Dynamics

Studies of dynamical phenomena remain relatively scarce compared to our current understanding of equilibrium properties. Most existing work has focused on charging dynamics, whereas comparatively few studies address fundamental transport properties, such as ion diffusivities within the pores. Moreover, the majority of experimental investigations to date have concentrated on carbon-based electrodes rather than on ionically low-dimensional pores as defined in this Focus Review. Such materials are inherently disordered and, in addition, can exhibit an interplay between ionic and electronic contributions, the separation of which has not yet been systematically investigated (Section 4.3.3).

#### 4.3.1 In-Pore Ion Mobility

Figure 4a presents MD simulation results demonstrating that ion diffusivity in 2D pores can vary by several orders of magnitude depending on the surface charge density,^6^ which is proportional to the applied cell voltage. This remarkable behavior was attributed to charge-induced ordering and disordering of ions within the pore. At low surface charge, confined ions form a quasi-ordered, nearly electroneutral 2D ionic structure that strongly suppresses ion mobility, yielding diffusivities up to 2 orders of magnitude lower than in the bulk electrolyte. As the surface charge increases, co-ions are progressively expelled from the pore while counterions are adsorbed to compensate the additional surface charge. This process disrupts the ordered ionic arrangement, leading to a pronounced enhancement of ion diffusivity, which can exceed the bulk value by up to an order of magnitude. Upon further increase of the surface charge, the pore becomes nearly depleted of co-ions, and the remaining counterions again organize into a quasi-ordered 2D structure, resulting in a renewed decrease in ion diffusivity. Although these results (Figure 4a) were obtained for a model ionic fluid composed of equally sized cations and anions—conditions that favor ordering under 2D confinement—qualitatively similar behavior has been observed in atomistic simulations of the confined ionic liquid [EMIM]­[TFSI].^83^

**Figure 4:**
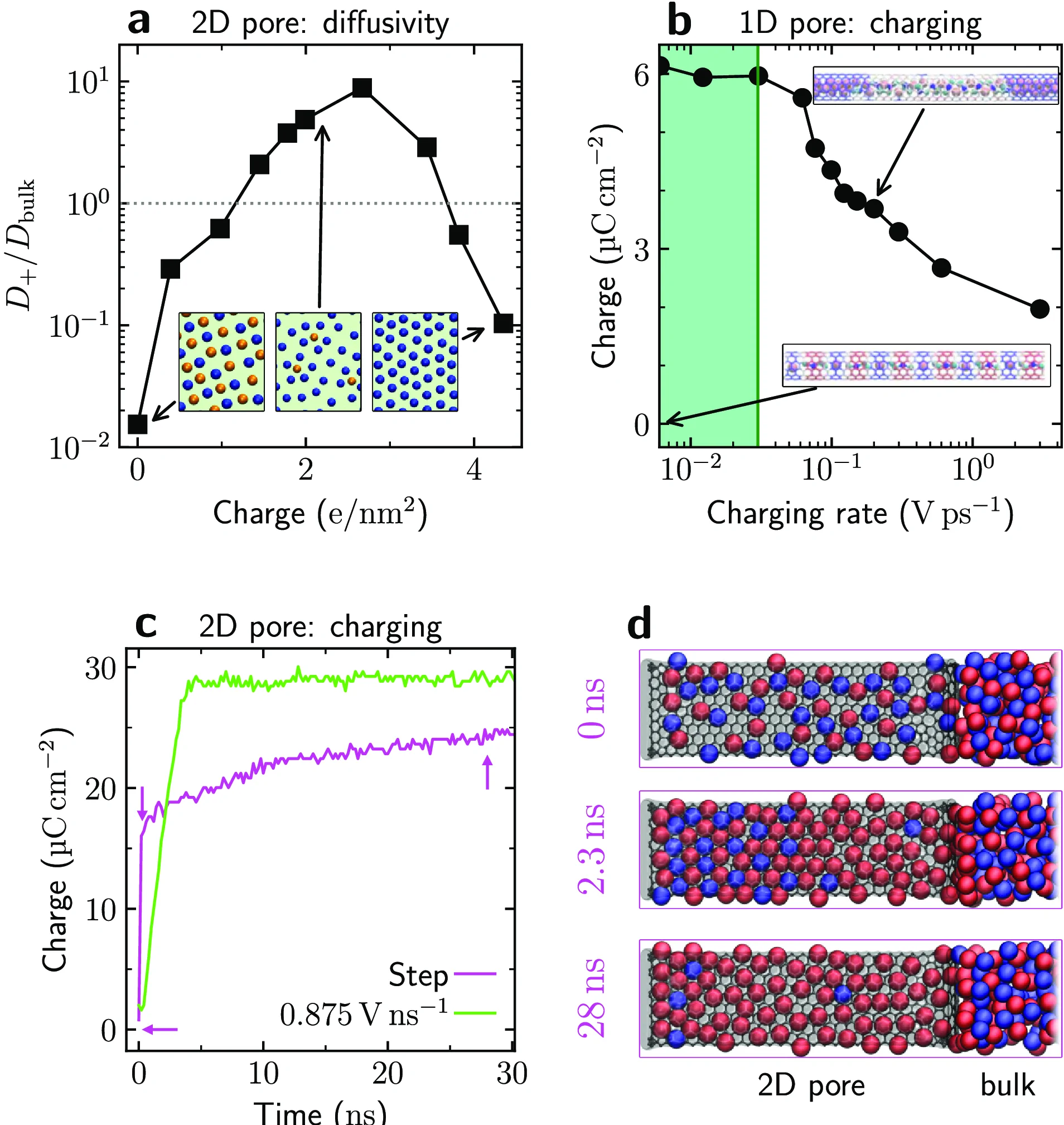
**Ionically low-dimensional ionotronics: dynamics.** (**a**) Ion diffusivity in 2D pores. The insets show representative ion configurations. Data and snapshots from ref 6. Copyright 2014 Springer Nature Limited. (**b**) Charge accumulated in a 1D pore of diameter *w* = 0.81 nm as a function of the rate of voltage application for [EMIM]­[BF_4_] ionic liquid. The insets show snapshots of uncharged and partially charged pores. The green area indicates the charging rates that ensure complete charging. Data and snapshots from ref 82. Copyright 2016 American Chemical Society. (**c**) Charge accumulated in a 2D pore as a function of time during step-voltage charging and under slow voltage ramp that avoid pore clogging. Ions are modeled as spherical charged particles with a diameter of 0.5 nm. The pore width is 0.6 nm. The arrows indicate the simulation times corresponding to the snapshots shown in panel (d). (**d**) Snapshots from MD simulations at the times indicated in panel (c). Data and snapshots from ref 9. Copyright 2018 American Chemical Society.

#### 4.3.2 Charging Dynamics

In addition to ionic structuring, the strong confinement imposed by low-dimensional pores can lead to ion crowding either within the pore or at its entrance during charging. Such pore clogging has been observed in both 1D and 2D confinements and results in markedly sluggish charging dynamics. These slow dynamics can, however, be mitigated by carefully tuning the rate of voltage application.^9,82^ Figure 4b shows MD results for the charging of a 1D pore of diameter 0.81 nm filled with the [EMIM]­[BF_4_] ionic liquid.^82^ The simulations reveal the formation of long-lived electroneutral regions inside the pore, which hinder complete charging on the simulation time scale. Notably, these regions shrink and eventually disappear as the voltage sweep rate is reduced.

Simulations of charging 2D pores revealed that pore clogging is a kinetic effect caused by the potential-driven adsorption of counterions during the initial stage of charging, which hinders the subsequent diffusion of co-ions out of the pore.^9^ Figure 4c–d illustrates the temporal evolution of the accumulated charge together with representative ion configurations following the abrupt application of a cell voltage across two slit nanopores. During the first ∼ 2.28 ns, counterions rapidly accumulate near the pore entrances, compressing the initially electroneutral ionic fluid within the pores. Consequently, the subsequent charging process becomes sluggish, being governed by the slow desorption of co-ions from this congested region. In contrast, gradually increasing the cell voltage can completely suppress pore clogging and dramatically accelerate charging—a strategy aptly termed “charge me slowly, I am in a hurry”.^9^ These simulations further revealed the existence of an optimal, voltage-dependent sweep rate, *k*_opt_, that minimizes the overall charging time (green line in Figure 4c). Notably, *k*_opt_ scales as 1/ l ^2^, where l denotes the pore length or electrode thickness.

Another strategy to accelerate charging is to render pores ionophobic, such that they are nearly free of ions at zero cell voltage. Simulations indicate that ionophobicity can enhance nanopore charging rates even by an order of magnitude.^6^ Experimentally, fluorination of activated carbons has been shown to increase ionophobicity and markedly improve capacitance at high scan rates.^83^ However, fluorination is also expected to alter the electronic properties of carbon—which already deviates from ideal metallic behavior (Section 5)—potentially affecting charging dynamics independently of ionophobicity. The role of these electronic effects in the observed enhancement remains unexplored.

#### 4.3.3 Low-Dimensional versus 3D Microporous Electrodes

Simulations of ion dynamics in 3D microporous electrodes—exemplified by a CDC structure with the electronic response modeled as that of an ideal metal—have shown that ion mobility depends markedly on the electrode charging state, similar to 2D pores (Figure 1a). In contrast to 2D pores, however, ion diffusivity exhibits a maximum close to zero cell voltage (zero charge) and decreases monotonically as the voltage increases in either the positive or negative direction.^84^ This behavior is likely due to counterion adsorption being the dominant charging mechanism, which increases the ion concentration within the pores and thereby reduces ionic mobility. NMR measurements on CDC electrodes revealed a much weaker voltage dependence,^85^ likely because they probe ion transport on millisecond time scales, over which the measured dynamics are averaged across the 3D pore network rather than reflecting local in-pore motion. These differences stem from the intrinsically disordered nature of the 3D pore structure, which prevents the formation of quasi-ordered ionic states that control ion mobility under 2D confinement. This distinction highlights a fundamental difference between 3D microporous electrodes and ionically low-dimensional systems.

Although charging dynamics of 3D microporous electrodes have been extensively studied—including through MD simulations^84,86^ —a systematic comparison with ionically low-dimensional electrodes remains lacking.

#### 4.3.4 Noise and Fluctuations in Ion Transport

Ion transport within or through nanoscale pores is intrinsically stochastic, leading to measurable current fluctuations. In sensing applications, such as DNA sequencing, the noise-to-signal ratio is a key performance metric, which has driven extensive research on biological (protein-based) nanopores that are typically nonconductive. Solid-state nanopores offer advantages including mechanical stability, tunable geometry, and facile device integration, and have also been widely studied;^87^ while most are electronically insulating, conductive nanopores based on materials such as graphene have also been considered.^88^

Current noise can be decomposed in the frequency (*f*) domain into four main contributions:^87^ pink noise (power spectra density, PSD ∝ 1/*f* ^α^, with α ≈ 1, hence also called 1/*f* noise), white noise (frequency-independent), dielectric noise (due to polarization losses), and amplifier noise (PSD ∝ *f* ^2^). In low-dimensional ionotronic systems, the PSD behavior provides direct insight into microscopic ion dynamics and ion–surface interactions. Unlike meso- or macroscopic electrochemical systems, extreme confinement amplifies the roles of surface charge, ion adsorption–desorption kinetics, dielectric polarization, and low-dimensional transport effects, resulting in nontrivial noise spectra. In particular, low-frequency 1/*f* noise is commonly associated with surface-charge and access-resistance fluctuations, whereas dielectric noise reflects polarization dynamics within the confining material.^88^ Recent studies of graphene nanopores^89^ demonstrated that reversible adsorption–desorption of ions at the pore walls is the dominant source of 1/*f* noise, whose amplitude can be tuned over several orders of magnitude. Notably, trace amounts of multivalent ions (0.1% of total concentration) could strongly enhance fluctuations, while high-dielectric solvents suppressed noise by weakening ion–surface interactions.^89^

These observations identify current noise as a sensitive probe of ion–surface coupling and interfacial dynamics in low-dimensional ionotronic systems; however, systematic studies of noise in such systems remain relatively scarce.

### 4.4 Electrolyte-Specific Effects

The effects discussed so far, such as superionic-state interactions (Section 4.1), are relatively generic and, in principle, applicable to a broad range of electrolytes, including ionic liquids as well as aqueous and other solvent-based systems. However, as noted Section 4.1, these interactions can depend sensitively on ion type and on the presence of solvent, in particular through their influence on dielectric response. In the following, we discuss electrolyte-specific effects, including ion valency, solvation, and in-pore ionic concentration.

#### 4.4.1 In-Pore Ion Concentration

For low-dimensional electrode materials immersed in a bulk electrolyte or in contact with an electrolyte reservoir (e.g., electrolyte contained within the separator of a supercapacitor; cf. Figure 6a), the in-pore ion concentration is determined by thermodynamic equilibrium with the bulk electrolyte. This equilibrium is governed by several competing effects that depend on both the electrolyte composition and pore properties, including direct ion–wall interactions, ion charge–image-charge attraction (Section 4.1.1), and (partial) ion desolvation upon pore entry, as low-dimensional confinement cannot accommodate the ion’s full solvation shell. The extent of desolvation generally increases with increasing confinement, making steric and solvation effects key factors limiting ion uptake in low-dimensional geometries. In contrast, image-charge attraction lowers the electrostatic energy of ions inside the pore (Section 4.1.1) and thereby promotes pore filling. For solvent-based electrolytes, increasing the bulk ion concentration typically also enhances the in-pore ion concentration. Simulations show that, as the bulk—and consequently the in-pore—ion concentration increases, the behavior of confined ions becomes increasingly dominated by steric interactions. This is reflected in the growing similarity of the ionic structure and diffusivities observed in conducting and nonconducting pores.^90^

Although a complete quantitative description of ion adsorption in low-dimensional pores remains unavailable, existing experiments and molecular simulations suggest that such pores are generally ionophilic,^91^ except when the pore dimensions become smaller than or closely comparable to the ion size. From an application perspective, deliberately designing ionophobic pores is particularly attractive, as such systems have been predicted to circumvent the power–energy trade-off in supercapacitors^6,77^ and may enhance the efficiency of capacitive desalination. Despite several promising experimental reports,^92–94^ unambiguous experimental evidence of ionophobic behavior in low-dimensional pores—together with clear demonstrations of the associated performance improvements and scalability toward industrially relevant systems—remains lacking (see ref 95 for a more detailed discussion).

#### 4.4.2 Multivalent Ions

Fundamentally, multivalent ions under low-dimensional confinement exhibit physics qualitatively similar to that of monovalent ions. In particular, the superionic-state interactions discussed in Section 4.1 remain applicable, with ion valencies *Z*_α_ entering explicitly into eqs 1 and 2. However, although interionic interactions are screened by low-dimensional conducting confinement, they become stronger with increasing ion valency. Consequently, disrupting ionic complexes within low-dimensional pores and bringing counterions into close proximity requires more energy, leading to higher applied potentials to achieve effects comparable to those observed for monovalent ions. The ion charge–image-charge attraction to the pore walls also increases with ion valency, thereby promoting pore filling. In contrast, desolvation of multivalent ions is typically more energetically costly because of their stronger solvation shells, reducing their tendency to enter low-dimensional pores. At the same time, the larger ionic charge allows more charge to be stored per ion, potentially increasing the stored energy density proportionally. The resulting storage capacity therefore reflects a balance between these competing effects. Simulations indicate that the net effect is generally positive, with both capacitance and stored energy density increasing with ion valency under low-dimensional confinement.^96^

Experimentally, multivalent ions have so far been explored primarily as alternatives to Li^+^-based batteries, particularly using calcium (Ca^2+^)^97^ and magnesium (Mg^2+^)^98^ ions, which are among the most abundant metallic elements in the Earth’s crust. Calcium ions have also been investigated for capacitive energy storage in mixtures of protic and aprotic ionic liquids with propylene carbonate,^99^ demonstrating favorable stability, ion transport, and capacitive behavior. However, these studies employed activated carbon electrodes rather than structurally well-defined low-dimensional systems.

Overall, despite recent progress^96,99,100^ —particularly in the context of battery research^97,98^ —the role of ion valency in low-dimensional ionotronics remains insufficiently explored. Further theoretical, computational, and experimental studies are needed to establish a more unified understanding of valency effects, their benefits and practical applicability.

#### 4.4.3 Solvent

Solvents can influence both equilibrium properties and ion dynamics in ionotronic systems. As noted in Section 4.2.1, solvent effects can reduce or even suppress the anomalous enhancement of capacitance in subnanometer pores.^76^ Polar solvents may additionally contribute directly to the capacitance, with the magnitude of this contribution governed by the solvent dipole moment,^101^ while electrode-philic solvents can render pores effectively ionophobic,^92,102^ thereby enhancing energy storage at elevated voltages.^77,103^ The extent of ion solvation and the chemical nature of the solvent can also affect ion rearrangement and charge storage. For instance, efficient desolvation of Li^+^ in carbonate-based electrolytes can substantially increase charge storage compared to nitrile- or sulfoxide-based solvents.^44^ Moreover, when pore sizes approach or fall below the hydrated ion diameter, desolvation energies become the dominant factor governing electrosorption and selectivity, particularly in subnanometer pores used for capacitive deionization.^104^

Adding solvent to room-temperature ionic liquids can enhance ion mobility and bulk electrolyte conductivity.^105^ Consistent with this trend, Burt et al.^106^ observed enhanced ion dynamics upon adding acetonitrile to [EMIM]­[BF_4_], although the equilibrium capacitance was only weakly affected. While this study focused on CDC electrodes—yet modeling the electronic response as that of an ideal metal—similar solvent-induced enhancements are expected in low-dimensional pores. Beyond in-pore dynamics, ion transport through angstrom-scale slits depends sensitively on hydrated ion size, highlighting the intricate interplay between confinement and solvation.^107^ In addition, membrane surface charge can modulate local solvent organization and ion distributions, shifting the ionic charge center closer to the pore wall. This strengthens ion–wall interactions and modifies frictional dissipation during transport,^108^ thereby enabling electrically tunable and selective ion permeation in membranes.^109,110^

## 5 Electronically Low-Dimensional Systems

Electronically low-dimensional electrodes do not provide narrow confinements for ions. Instead, atomically thin pore walls alter the electronic properties of the electrode compared to its 3D counterpart, influencing the electrochemical behavior of the electrode–electrolyte system as a whole. Such effects were first discussed by Gerischer^111^ for graphite, which also exhibits electronic properties qualitatively distinct from metals. While various electrode material featuring thin walls have been developed (Section 3 and Figure 2), we discuss here graphene and carbon nanotubes (CNTs) as most extensively studied representatives of 2D and 1D electronically low-dimensional electrodes. Graphene and CNTs have been particularly well investigated theoretically, yielding reliable analytical formulas for computing their electronic properties. This robust theoretical framework will help us demonstrate their key characteristics.

### 5.1 Graphene as 2D Electrodes

Defect-free, pristine graphene is characterized by a low density of states near the Fermi level, which results in a low number of charge carriers and, consequently, a low *quantum capacitance*, *C*_q_. Analytical calculations^112^ yield the following elegant expression for *C*_q_:

6 $$C_{q}\left(u_{q}\right)=\frac{2e^{2}k_{B}T}{\pi \hbar v_{F}}\ln\left[2\left(1+\cosh\frac{eu_{q}}{k_{B}T}\right)\right]$$

where *u*_q_ is the gate voltage, *ℏ* the reduced Planck constant, and *v*_*F*_ ≈ 10^8^cm/s is the Fermi velocity of charge carries in graphene. At zero gate voltage and a temperature of *T* = 300 K, eq 6 gives *C*_q_ ≈ 0.42 μF/cm^2^, which is significantly lower than typical values of electrical double-layer capacitance (see e.g. Figure 3d,e). This contrasts with 3D bulk metals and some metallic MXenes,^38^ which possess a virtually infinite quantum capacitance. In Figure 5a, we plot *C*_q_ as a function of *u*_q_ obtained by using eq 6. *C*_q_ is symmetric relative to *u*_q_ = 0, where it shows a minimum, and steadily increases with increasing the absolute value of *u*_q_. This behavior is supported by robust quantum density functional calculations^113^ and experiments.^114^ Bilayer and multilayer graphene (except for twisted bilayer^115^) typically have similar properties but exhibits a progressive increase in capacitance with the number of layers.^114^

An electrolyte interfacing with graphene (or any other electrode) can be viewed as an “ionic capacitor” connected in series with an “electronic capacitor”. The total capacitance of this system is

7 $$\frac{1}{C\left(u\right)}=\frac{1}{C_{q}\left(u_{q}\right)}+\frac{1}{C_{IL}\left(u-u_{q}\right)}$$

where *C*_IL_(*u*_IL_) denotes the ionic capacitance at potential difference *u*_IL_ between the electrode and the bulk electrolyte (shown in Figure 3c,d,e). Note that *C*_q_ = *∞* and *u*_q_ = 0 for 3D ideal metal, yielding *C* = *C*_IL_.

To showcase the impact of a finite *C*_q_ on the overall capacitance, we used eq 6 for *C*_q_(*u*_q_) shown in Figure 5a alongside an analytical equation for *C*_IL_(*u*_IL_) derived from the lattice gas model of electrolytes.^116^ While this model does not provide accurate quantitative predictions, it captures the essential physics and characteristic shapes of electrical double layer capacitance.^116^ Figure 5b compares the capacitance between an ideal metal electrode and a graphene electrode, indicating that the graphene electrode decreases the capacitance across all applied potentials. The most notable reduction occurs around zero volts, where even the capacitance shape changes to follows the shape of the quantum capacitance. This behavior aligns well with experimental observations, as demonstrated in Figure 5c for a 6 M KOH electrolyte at a graphene electrode.^117^

### 5.2 Carbon Nanotubes as 1D Electrodes

CNTs represent an important class of electrode materials with electronic properties exhibiting quasi 1D behavior. A CNT can be theoretically constructed from a cut graphene sheet rolled into a cylinder and is characterized by two numbers (chirality indices), indicating the cutting plane in terms of two linearly independent basis vectors. Due to periodic boundary conditions along the circumference, the electron motion in a CNT is quantized and becomes effectively one-dimensional in the direction of the CNT central axis. Depending on their chirality indices, CNTs can be metallic or semiconducting, a direct consequence of the zero-gap band structure of graphene; the specific cutting and wrapping of the graphene plane into a cylinder shifts the bands, either increasing the band gap or making the bands overlap.

In Figure 5d, we depict quantum capacitance (*C*_q_) of metallic and semiconducting CNTs of nearly identical radii. *C*_q_ follows a qualitatively similar pattern in both cases but vanishes for the semiconducting CNT within the first subband, determined by the so-called van Hove singularities. This difference between metallic and semiconducting CNTs manifests itself in the electrochemical behavior of the CNT-electrolyte system. Figure 5e shows that the low quantum capacitance of both CNTs reduces the overall capacitive performance compared to a metallic cylinder of the same size. Interestingly, while the semiconducting CNT exhibits zero capacitance within the first subband, it outperforms the metallic CNT as the potential increases. This behavior follows from a higher quantum capacitance of the semiconducting CNT at elevated gate voltages (Figure 5d). Figure 5f shows experimental results for semiconducting CNTs of similar radii and 1 M aqueous KCl electrolyte, demonstrating similar features as predicted theoretically.

**Figure 5:**
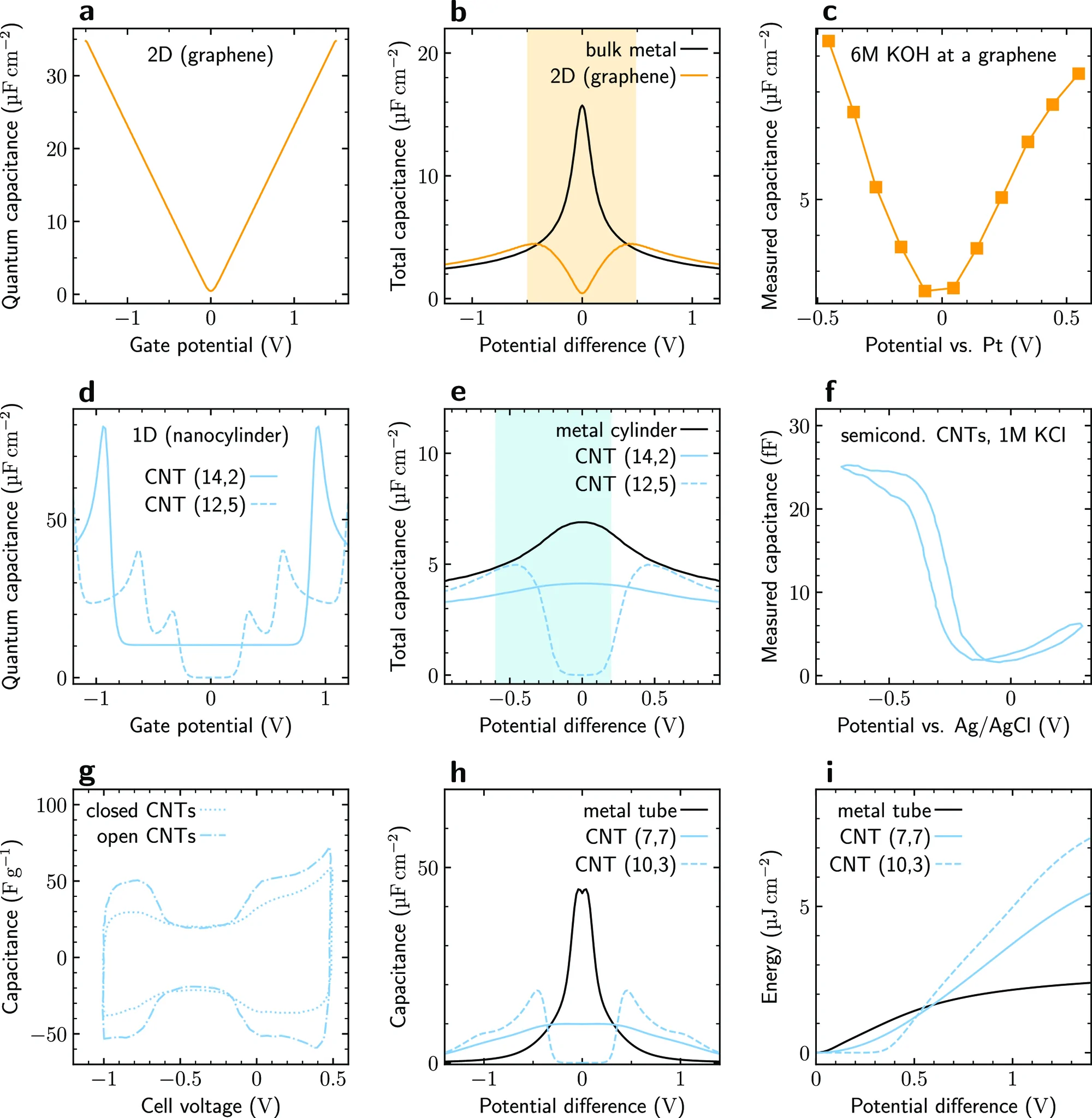
**Effect of electronic low-dimensionality on ionotronic systems.** (**a**) Quantum capacitance *C*_q_ of graphene as a function of gate voltage (eq 6). (**b**) Total capacitance *C* of a 3D ideally metallic flat electrode and a 2D graphene electrode computed using eq 7. For an ideal metal, quantum capacitance *C*_q_ = *∞* and therefore the total capacitance *C* = *C*_IL_, where *C*_IL_ is the ionic capacitance. *C*_IL_ was calculated using the analytical expression derived from the lattice-gas model in ref 116 (temperature *T* = 300 K, relative dielectric permittivity ε_r_ = 20, electrolyte concentration 3 M, and parameter γ = 0.45). The orange box indicates the voltage range explored experimentally in ref 117 and shown in panel (c). (**c**) Total capacitance of 6 M aqueous KOH at a graphene electrode. Data from ref 117. (**d**) Quantum capacitance of semiconducting and metallic CNTs with chiral indices (12,5) and (14,2), respectively. The CNT diameter is approximately 1.2 nm in both cases. Data calculated using the analytical expressions of ref 118. (**e**) Total capacitance of an ideally metallic cylinder and of semiconducting and metallic CNTs. The *C*_IL_ data are from ref 119 (parameters as in panel (f)). The blue box indicates the voltage range explored experimentally in ref 119 and shown in panel (f). (**f**) Total capacitance of 1 M aqueous KCl in closed semiconducting CNTs. Data from ref 119. (**g**) Cyclic voltammograms of open and closed CNTs with diameters ranging from 1.47 to 1.67 nm. Data from ref 120. (**h**) Capacitance and (**i**) stored energy as functions of the potential difference between the nanotube and the bulk electrolyte for an open ideally metallic nanotube and for metallic and semiconducting CNTs with chiral indices (7,7) and (10,2), respectively. The nanotube diameter is approximately 0.9 nm in all cases. Data from ref 121.

### 5.3 Cross Wall, Interpore Communication

An intriguing aspect of electronically low-dimensional electrodes is their partial transparency to electrostatic and other interactions, which can lead to correlated behavior of ions and solvent molecules on opposite sides of atomically thin pore walls. Molecular simulations revealed that like-charged ions on opposite sides of a conducting wall can become positively correlated, i.e., they tend to align across the wall.^122^ Similarly, quantum density functional theory (DFT) calculations demonstrated that two like-charged ions may effectively attract one another when located inside and outside a single-wall CNT.^123^ Such cross-wall correlations arise because ions on opposite sides of a thin wall induce countercharges in the conducting material, which mediate an effective attraction between them. Atomically thin walls have also been shown to exhibit wetting transparency,^124,125^ cross-wall electron transfer,^126^ and flow tunneling, whereby two liquids separated by an atomically thin wall exchange momentum.^127^

These phenomena can strongly influence charge storage and ion dynamics in electrodes containing atomically thin low-dimensional walls. Model calculations^128^ indicated that positive cross-wall ionic correlations can enhance accumulated charge at moderate voltages and even induce phase-transition-like charging behavior. We recall that such abrupt charging transitions can be observable only in structurally well-defined low-dimensional electrodes, since structural disorder and pore-size dispersion tend to smear out their signatures (Section 4.2.4 and Figure 3d). The availability of 2D materials with wall thicknesses ranging from a single atomic layer (graphene) to several atomic layers (M_5_X_4_T_*x*_ MXenes) should enable systematic computational and experimental investigations of these effects, including determination of the wall thickness and electronic density required to fully suppress cross-wall ionic interactions.

Overall, pore-wall thickness provides an additional parameter for tuning and optimizing the behavior of low-dimensional ionotronic systems. However, these effects remain insufficiently explored. In particular, the possibility of negatively correlated cross-wall ionic interactions—which may enhance pore ionophobicity and thereby improve energy storage^128^—as well as the role of wall transparency in redox processes, pore ionophilicity, and the influence of flow tunneling on charging dynamics, warrant further investigation.

## 6 Electronically and Ionicaly Low-Dimensional Systems

The interplay between ionic and electronic low-dimensionality has been explored experimentally with CNTs, which can be synthesized either with closed ends or in an open configuration that permits ion ingress. Results shown in Figure 5g demonstrate that opening CNTs has little effect on capacitance at low cell voltages but leads to a pronounced enhancement as the voltage increases. This behavior is likely governed by entropic barriers that hinder ion entry into the nanotubes at low polarization (cf. Figure 3d). As the applied potential increases, these barriers are progressively overcome, enabling ion insertion and thereby enhancing the capacitance.

In Figure 5h, we compare the total capacitance (*C*) of an ideally metallic nanotube (i.e., with the electronic response of a 3D ideal metal) with that of metallic and semiconducting CNTs of similar size. At low potential differences relative to the bulk electrolyte, metallic CNTs exhibit a reduced capacitance compared with the metal nanotube, while semiconducting CNTs display an almost vanishing capacitance, closely resembling the behavior of closed nanotubes shown in Figure 5e. At higher potentials, the trend changes qualitatively: the capacitance of the metallic nanotube decreases markedly, whereas that of the CNTs remains higher, particularly for semiconducting CNTs, which eventually surpass both metallic CNTs and ideally metallic nanotubes.

This effect originates from the low quantum capacitance of CNTs. Ideally metallic nanotubes become saturated with counterions already at relatively low applied potentials, thereby suppressing further charge accumulation. In contrast, the finite and comparatively low quantum capacitance of CNTs shifts this saturation to higher voltages, enhancing the total capacitance in the high-voltage regime.^121^ In this sense, a smaller quantum capacitance can paradoxically result in a larger overall capacitance—a “less is more” effect^121^ with important implications for stored energy density:

8 $$E\left(u\right)=\int_{0}^{u}vC\left(v\right)dv$$

Figure 5i illustrates that, except at relatively low voltages, semiconducting CNTs (low *C*_q_) exhibit the highest stored energy density, whereas ideally metallic nanotubes (infinite *C*_q_) display the lowest. Similar effects have also been reported for slit pores formed by graphene planes.^129^

While low-dimensional electrodes with low *C*_q_ can offer advantages for energy storage, their limited electronic conductivity may also lead to energy dissipation. This issue is particularly relevant for semiconducting CNTs, where resistive losses can generate waste heat, necessitating effective thermal management and cooling strategies. Despite these potential drawbacks, low quantum capacitance presents an intriguing opportunity for enhancing energy storage capabilities, highlighting the need for systematic investigations of the interplay between ionic and electronic low-dimensionality. Further research is therefore essential to understand how these factors influence device performance, particularly in materials such as MXenes, where these effects remain largely unexplored.

## 7 Emergent Applications

We now outline key areas and phenomena where low-dimensional ionotronics can enhance performance or enable novel functionalities. In many cases, low-dimensional electrodes offer advantages due to their tunable structures, which can be tailored to specific electrolytes and applications. Moreover, they allow for the exploitation of new effects such as in-pore phase transitions,^4,8^ breaking of the Coulombic ordering,^130,131^ and the formation of ordered ionic structures in 2D pores,^132^ which do not exist in conventional materials.

### 7.1 Energy Storage

Energy storage is arguably the most straightforward application of low-dimensional ionotronics, readily integrable in existing devices such as electrical double-layer capacitors (EDLCs), pseudocapacitors, and batteries. In batteries and pseudocapacitors, electrical energy is stored and released through Faradaic reactions. The use of low-dimensional materials enables high tunability in band structure, surface charge, and ion transport. Incorporating materials such as MXenes and graphene into battery electrodes has led to enhanced performance, primarily due to their high electrical conductivity and large surface area, which facilitate efficient electron transport and ion diffusion.^138,139^

An EDLC stores energy through voltage-induced electrosorption of ionic charge (Figure 6a). Because capacitive energy storage does not involve chemical reactions and its charge–discharge kinetics are governed primarily by ion transport, EDLCs exhibit high power densities and excellent cyclability; however, their energy densities remain lower than those of batteries. EDLCs are therefore widely employed in applications requiring rapid energy delivery or harvesting, such as buffering intermittent renewable energy sources or providing backup power to prevent blackouts.

**Figure 6:**
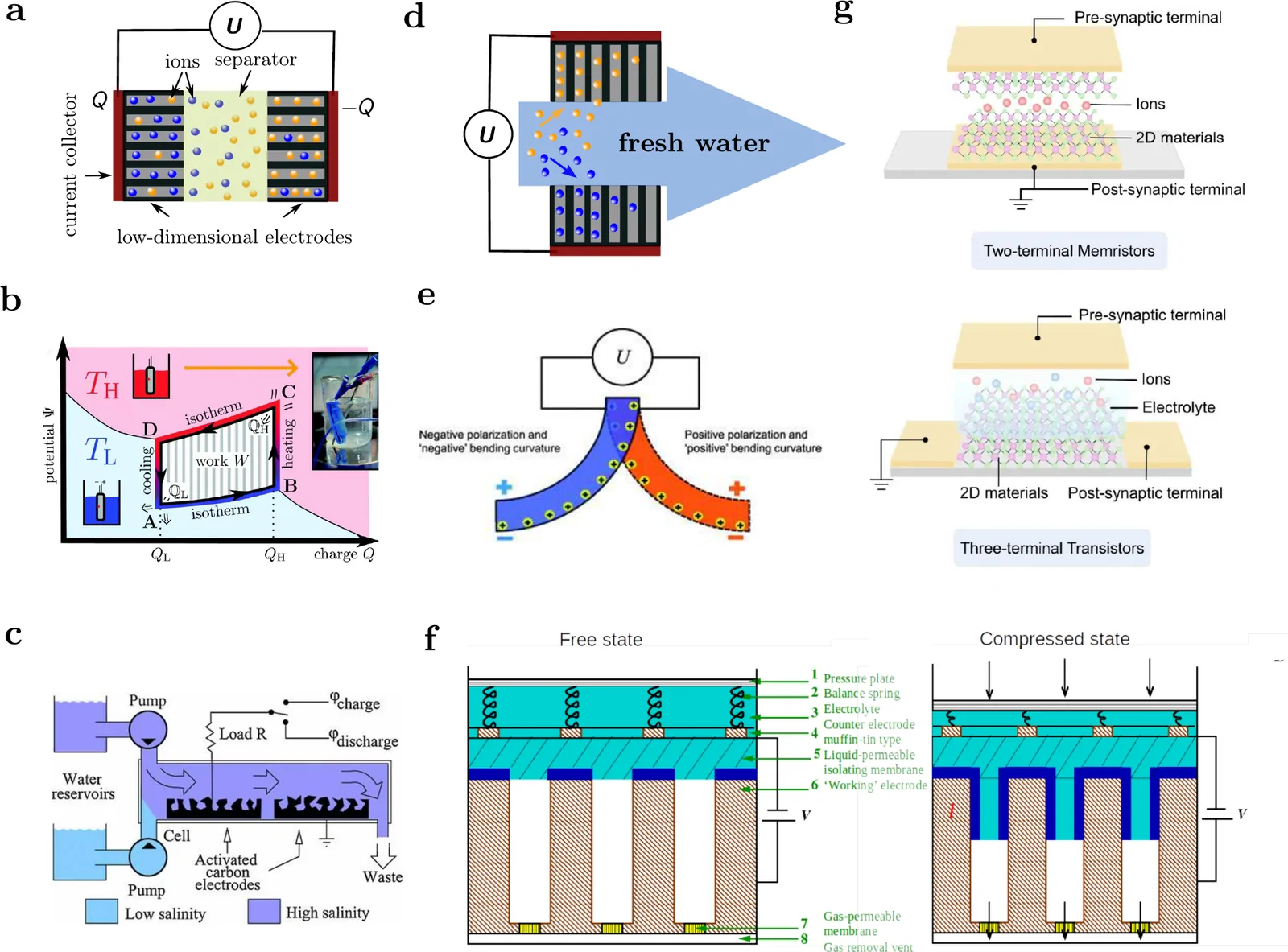
**Emerging applications of low-dimensional ionotronic systems.** (**a**) Energy storage using electrical double-layer capacitors (EDLCs). Two electrolyte-filled low-dimensional electrodes connected to current collectors are separated by an ion-permeable membrane to prevent short-circuiting. Energy is stored through reversible electrosorption of ionic charge within low-dimensional pores. (**b**) Conversion of waste heat into electricity via a Carnot-like cycle, achieved by isothermal charging and discharging of an EDLC at high and low temperatures. Reproduced from ref 133. Available under Creative Commons CC BY 3.0. Copyright 2015 Härtel et al. (**c**) Blue-energy harvesting using CAPMIX technology, where energy is extracted through cyclic charging and discharging of EDLCs in electrolytes with low and high salinities. Reproduced with permission from ref 134. Copyright 2009 American Physical Society. (**d**) Capacitive deionization (CDI) for water desalination, in which ions from brackish water are electrosorbed into EDLC pores during charging and released during discharging. (**e**) Capacitive electroactuation using EDLCs with highly size-asymmetric ions and flexible electrodes for controlled motion in smart devices. The bending direction of the EDLC depends on the applied cell voltage. Reproduced with permission from ref 135. Copyright 2013 Royal Society of Chemistry (Great Britain) (**f**) Electrochemical generator driven by pressure variations, producing current through walking-induced modulation of the electrode–electrolyte contact area. Adopted with permission from ref 136. Copyright 2016 Institute of Physics (Great Britain). (**g**) Two-terminal and three-terminal neuromorphic ionotronic devices incorporating pre- and postsynaptic-like terminals (and a gate/control terminal in the three-terminal configuration), where ion transport and electrolyte conductivity are modulated by low-dimensional electrode materials. Reproduced with permission from ref 137. Copyright 2025 American Chemical Society.

Several experimental studies have explored EDLCs based on low-dimensional electrodes, including graphene^140^ and MXenes,^141^ demonstrating enhanced energy storage and improved charging dynamics. Nevertheless, a clear experimental realization and practical implementation of monodisperse 1D or 2D ionotronic systems for energy storage remain elusive. In particular, the experimental demonstration of the “less-is-more” concept—where low quantum capacitance enhances energy storage^121^ (Figure 5h,i)—has not yet been achieved. Similarly, in-pore phase transitions,^4,8,132^ such as those illustrated in Figure 3e, have also not yet been observed experimentally (see Section 7.10 for a discussion of their potential applications).

In pseudocapacitors, where both EDLC charge storage and Faradaic reactions contribute to overall capacitance, low-dimensional materials with highly tunable surface chemistry and structure can facilitate fast and reversible redox reactions, resulting in high capacitance and energy density. Beyond conventional EDLC-based energy storage, MXenes with 2D nanoconfinement exhibit pseudocapacitive behavior in acidic and organic electrolytes. This behavior arises from surface termination redox reactions or orbital overlap between cations and MXene layers.^47,142^

### 7.2 EDLCs: Disordered Microporous or Ordered Low-Dimensional Electrodes?

No consensus has emerged in the literature on whether disordered microporous electrodes or ordered low-dimensional electrodes provide higher capacitance and stored energy density in EDLCs. For 2D electrodes, our previous work demonstrated^81^ the existence of an optimal pore width that maximizes the stored energy density at a given cell voltage, suggesting that monodisperse low-dimensional electrodes can potentially outperform disordered porous materials. This view has recently been challenged. Simulations comparing charging of CDCs and 2D electrodes suggested that CDCs may exhibit better performance;^143^ however, it remains unclear whether the investigated 2D electrodes represented the optimal pore configuration. More recently, experiments reported that more disordered carbons exhibited higher capacitances;^73^ however, no direct comparison with low-dimensional electrodes has yet been performed.

In disordered porous electrodes, some regions contribute more strongly to the overall capacitance than others. Although identifying and isolating such regions experimentally may be challenging, constructing electrodes composed exclusively of favorable local pore environments could, in principle, enhance charge storage and capacitance. Potential limitations associated with slower charging dynamics can be mitigated through hierarchical architectures or by employing the “charge-me-slowly” strategy (Section 4.3.2 and Figure 4c). Thus, designing electrodes with controlled low-dimensional pore architectures may offer a route toward improved electrochemical performance. However, this potential has not yet been demonstrated explicitly through simulations or experiments, and the fundamental principles underlying the design of optimal low-dimensional electrodes remain to be established.

### 7.3 Harvesting Thermal Energy

With rising global energy demands and growing environmental concerns, efficient energy harvesting has become a critical challenge in transitioning from fossil fuels to clean, renewable energy sources and achieving sustainable energy independence. Low-dimensional materials can facilitate innovative solutions for advancing energy harvesting technologies. One promising approach is the recovery of waste heat, which is abundantly present in industry and everyday life, such as in high-performance computing centers, aircraft, laptops, and smartphones. For instance, converting temperature variations in a smartphone into electrical energy can enable self-recharging, akin to regenerative braking in vehicles, while also enhancing thermal management to extend device lifespan. Such heat-to-electricity converters (HECs) could leverage the temperature dependence of EDLC capacitance, analogous to a heat engine that utilizes the temperature dependence of pressure to convert thermal energy into mechanical work. Charge–discharge cycles in HECs correspond to the contraction–expansion cycles of a heat engine, with cell voltage (*u*) and accumulated charge (*Q*) playing roles analogous to pressure and volume, respectively. Figure 6b illustrates an HEC cycle consisting of isothermal charging and discharging and constant-charge heating and cooling. The work delivered by an HEC is given by

9 W=−∮udQ

as indicated by the shaded area in Figure 6b. Although HEC efficiencies are so far relatively low (below Carnot efficiencies),^133^ they provide opportunities for harvesting heat that would otherwise be dissipated to the environment.

The key challenges in advancing HECs lie in exploring the temperature dependence of nanopore charging, which remains poorly understood. Optimizing HECs requires maximizing the charge accumulation difference across the operating temperature range, which can be achieved by tailoring nanopore and electrolyte properties. The unique ability of low-dimensional electrodes to form monodisperse porous structures can enable more precise fine-tuning of HEC performance.^144^

### 7.4 Blue Energy

Another promising approach for sustainable energy harvesting involves exploiting capacitance variations with salinity. Salinity gradients are abundant in estuaries, where fresh water from rivers mixes with salty seawater, offering a vast and clean energy source known as blue energy. Traditional blue energy technologies rely on reverse electrodialysis (RED) and pressure retarded osmosis (PRO), both of which require costly membranes. In contrast, the so-called CAPMIX technology^134^ is membrane-less and operates by utilizing the salinity dependence of double-layer capacitance (however, some extensions also involve membranes^145^). This approach constitutes of four steps: (1) charging the electrodes in seawater; (2) replacing the saltwater with freshwater, which increases the electrode potential while keeping the accumulated charge constant by disconnecting the electrodes; (3) discharging the electrodes, and (4) replacing the freshwater back with seawater to complete the cycle (Figure 6c). As the energy required for charging is lower than the energy released during discharging, the complete cycle yields a net energy gain, given by eq 9.

A proof-of-principle device demonstrated a power output of ca. 7 mW/m^2^ of CDC electrodes per cycle.^134^ Current capabilities have reached hundreds of mW/m^2^, comparable to those of pressure retarded osmosis and reverse electrodialysis.^134,145,146^ While blue energy extraction has been demonstrated with MXenes used as a 2D membrane,^147^ CAPMIX technology with low dimensional electrodes remains unexplored. These electrodes could enhance harvesting by enabling accurate pore-size matching to seawater ions, increasing accumulated charge and thereby voltage rise in step 2 of the cycle. Additional challenges include minimizing ohmic losses and current leakage during the second extraction step, as well as reducing switching time.

### 7.5 Desalination and Ion Separation

The scarcity of fresh water in regions like the Middle East, Africa, and Australia has made water desalination increasingly important, with large-scale desalination plants already functioning globally. Energy sources abundant in these areas—particularly renewable energy like solar power—make compact, local, off-grid desalination systems feasible and appealing. While thermal-based, reverse osmosis (RO), and reverse electrodialysis (RE) are the three dominant desalination technologies today, capacitive-based methods are gaining momentum due to their distinct advantages, such as lowering costs, reducing environmental impact, and eliminating the need for expensive membranes and high-pressure pumps associated with pressure-driven methods. Capacitive desalination, more often referred to as capacitive deionization (CDI), operates as a reverse process to blue energy extraction, where brine water is passed through a supercapacitor with highly porous electrodes. Upon polarization, the cathode adsorbs cations while the anode adsorbs anions, producing water with lower salinity^148^ (Figure 6d).

The porous electrodes can be made from low-dimensional materials, such as graphene^149^ and MXenes.^150^ Narrow, ordered, and highly monodisperse pores in these electrodes can greatly enhance ion adsorption capacity, e.g., by minimizing the ion-exchange charging mechanism, which is detrimental to desalination efficiency. Ultranarrow 1D and 2D pores—which inhibit ion adsorption at zero electrode polarization (see 1D pores in Figure 3d)—can be especially beneficial for achieving this goal. Key challenges requiring further attention include developing a deeper understanding of ion transport in narrow pores and optimizing flow rates to enhance ion adsorption efficiency.

Unlike RO and RE, conventional CDI is limited to ion extraction and does not filter bacteria or colloidal particles. To overcome these limitations, desalination and filtration with low-dimensional materials can be done in an RO-like setup. For instance, brine water can be pumped through a graphene membrane with nanoscale pinholes, where the graphene also serves as an electrode. Polarizing the graphene negatively creates a barrier for anions, while adding consecutively a positively polarized graphene layer would impede the transport of cations. New engineering solutions must be developed to remove the blocked ions and optimize desalinated water production efficiently. The key physical challenges to resolve involve understanding the electrokinetics of ion transport through the graphene pinholes and the dynamics of the double-layer formation at the polarized graphene/solution interface.^151,152^

### 7.6 Electrochemical Actuation

Electrochemical actuation refers to the conversion of electrical energy into mechanical work or motion and has a wide range of applications, including artificial muscles, bionic robots, *in vivo* surgery devices, and other smart technologies. Capacitive (also called ionic) electroactuation operates by inducing shape or size changes in the electrodes through ion accumulations driven by polarization and ion size asymmetry. Such electroactuators consist of an electrolyte-permeable membrane sandwiched between two thin, flexible electrodes. Upon voltage application, larger ions preferentially accumulate at one electrode, causing the structure to bend and generate motion^153^ (Figure 6e). Due to their exceptional mechanical stability and flexibility, low-dimensional electrodes like CNTs, graphene,^154^ and MXnene^155^ have been explored for capacitive electroactuation. Moreover, recent publications report on MXene-graphene hybrid electrodes with the mechanical strength exceeding that of steel.^156^ These ultrastrong 2D materials with ions confined in 2D structures, capable of electrochemical charging, can be the game changers in robotics and actuation. The main challenges lie in understanding the coupling between the mechanical and electrochemical properties of low-dimensional electrodes and the kinetics of charging vs the mechanics of bending or expansion/contraction.

### 7.7 Reverse Electrochemical Actuation

Reverse actuation presents yet another opportunity for harnessing ubiquitous renewable energy sources. Similar to blue energy extraction, which operates as the reverse of capacitive desalination, electroactuation can also function in reverse to generate electricity from mechanical motion. This process relies on the periodic bending of a supercapacitor by external forces, inducing charge accumulation and changes in the electrode potential, thereby generating a net electrical current. This approach could enable energy recovery from natural or artificial movements, such as ocean waves, human motion, or structural vibrations, offering a promising pathway for self-powered devices and sustainable energy harvesting. These devices can have versatile applications, such as powering everyday-use items like smartphones or watches.

One of the most straightforward implementations is an electroactuator placed outdoors to capture wind energy through bending. Several other possibilities have been suggested, such as an “electrical shoe” or porous sole device^157^ that generates current by periodically altering the electrolyte-electrode contact area while walking (Figure 6f).

Unlike direct actuation, which focuses on generating large mechanical motion from small voltage applications, reverse actuation aims to achieve high currents from small mechanical motions, highlighting the fundamental difference in their optimization principles. Yet, the key challenge remains the same: to better understand the interplay between bending mechanics and charging dynamics.

### 7.8 Sensing

In addition to their use as peak power supplies for wireless data transmission in various sensing devices, supercapacitors can also function as sensors themselves, leveraging their electrochemical and electroactuation properties. For instance, supercapacitors can function as potentiostatic sensors for determining pH and ion concentration or as oxidative current-based sensors for detecting the monoamine level.^158^ Furthermore, the reverse electroactuation effect, which generates a current in response to mechanical deformation, can be utilized for an accurate pressure sensing or for detecting changes in gas or liquid flow.

Unlike reverse actuation, but similar to direct actuation, achieving sufficiently high currents in response to small mechanical deformations is crucial for ensuring high device sensitivity. Large *variations* in current induced by small mechanical changes are likewise desirable, as they can improve sensitivity and broaden the operational window of the device. In addition, the exceptional mechanical strength of 2D materials such as MXenes, graphene, and their composites^156^ may open new opportunities for high-pressure sensing applications.^159^

### 7.9 Neuromorphic Applications

Neuromorphic computing represents one of the most compelling applications of low-dimensional ionotronics. As conventional computing architectures increasingly suffer from limited bandwidth between physically separated processing and memory units (the so-called von Neumann bottleneck), the resulting power consumption and latency have become major challenges, particularly for artificial intelligence (AI) applications. Neuromorphic approaches offer a promising route toward overcoming these limitations by enabling low-power, highly parallel, and energy-efficient computation. Inspired by the brain’s seamless integration of memory and information processing, as well as its remarkable energy efficiency, ionotronic neuromorphic devices emulate synaptic functionality by using electronically driven ionic motion to modulate conductance states.

Among the various device architectures, two-terminal (memristive, i.e. resistive devices with memory^160,161^ ) and three-terminal (transistor-like) artificial synapses have emerged as particularly promising hardware elements for neuromorphic computing (Figure 6g). In these devices, the resistance of the active material between terminal electrodes—analogous to the synaptic cleft between pre- and postsynaptic neurons—depends on the history of applied electrical pulses, thereby enabling memory storage and synaptic plasticity. Such behavior can arise from a variety of physical mechanisms,^162^ including the formation and rupture of conductive filaments,^163^ ionic redistribution in chalcogenides,^164^ and polarization switching in ferroelectric layers.^165,166^ Another route toward memristive functionality exploits hysteretic ion transport through conically shaped nanopores, mimicking ionic transport in biological ion channels.^167,168^ In such systems, slow ionic redistribution and concentration polarization within the asymmetric pore geometry generate history-dependent ionic conductance and memristive behavior.^169,170^ More complex architectures, incorporating additional terminals or combinations of devices, can further enable Boolean logic operations, associative learning (such as Pavlovian conditioning), and other advanced neuromorphic functionalities.^171^

Low-dimensional materials offer exciting opportunities for neuromorphic device development. Under 2D conductive confinement, ions can form pairs and higher-order assemblies—so-called “Bjerrum polyelectrolytes”—which modify ionic conductivity by effectively changing the number of mobile charge carriers^172,173^ (such structures were first identified in Monte Carlo simulations by Dudka et al.,^130^ who termed them “ionic snakes,” but their implications for conductivity and neuromorphic functionality were not addressed). For high-frequency signals, the formation of these structures is too slow to follow the instantaneous electric field. Once formed, however, they may persist after field removal if their dissociation is slower than the field variation, thereby giving rise to memristive behavior.^172,173^

Atomic-scale ionic confinement in low-dimensional materials results in strong electrostatic coupling between electronic and ionic degrees of freedom, enabling low-voltage—and therefore low-energy—operation without compromising device functionality. Early demonstrations include graphene-based synapses in which the electronic conductance is modulated by the intercalation of Li^+^ ions between graphene layers.^174^ These devices successfully reproduced key synaptic functions, including excitatory and inhibitory responses, long-term potentiation and depression, and spike-timing-dependent plasticity, but operated with relatively modest energy efficiency (ca. 500 fJ per event) compared with biological synapses (∼1 fJ to 10 fJ). More recent 2D ionotronic synapses have achieved substantially improved performance, with reported energy consumptions approaching the biological range. Notable examples include tellurene-based artificial synaptic transistors operating at approximately 9 fJ per event^175^ and MoS_2_/h-BN/graphene floating-gate devices reaching 3.2 fJ per operation.^176^

Overall, low-dimensional—particularly 2D—ionotronic neuromorphic systems constitute a rapidly evolving field, encompassing a broad range of device architectures and fabrication strategies. Given the pace and diversity of developments, this topic warrants dedicated reviews; for comprehensive discussions, we refer the reader to recent articles.^137,177,178^

### 7.10 In-Pore Ionic Ordering and Phase Transformations

A distinct feature of 2D electrodes is their ability to induce phase transformations in confined electrolytes—an effect starkly contrasting with 1D and 3D porous electrodes. So far, in-pore 2D phase transformations have only been reported in theoretical^4,132^ and simulation^8^ studies. However, low-dimensional materials like MXenes may open new avenues for experimental investigations of phase-changing confined ionic fluids.

Beyond their fundamental scientific interest, particularly in connection with the celebrated Berezinskii–Kosterlitz–Thouless transition,^179^ in-pore (2D) phase transformations hold promise for novel practical applications. Phase-change materials (PCMs) have recently attracted significant attention for their potential in efficient thermal energy storage and conversion.^180^ These latent-heat storage systems exploit the enthalpy associated with reversible first-order transitions (such as liquid–gas or solid–liquid transitions) to achieve high thermal-storage densities within a narrow temperature range. Low-dimensional electrodes could be integrated into such systems, opening new opportunities for combined thermal and electrochemical energy storage.

Another promising direction for enhancing electrochemical energy storage is to promote ionic ordering in nonpolarized pores. Similar to the effects predicted for ionophobic pores,^6,77^ overcoming the free-energy barrier associated with disrupting this ordered state may substantially increase the stored energy density at elevated cell potentials.^95,132^

PCMs have also been explored in thermoelectric generators for converting thermal energy into electricity via the Soret (or electrolyte Seebeck) effect,^181,182^ whereby a temperature gradient between two electrodes generates an electric current.^183^ In such systems, the primary role of the PCM is to maintain or enhance the temperature difference across the generator, thereby improving its efficiency. For example, solid-to-liquid phase transitions in sodium–potassium alloy electrodes have been shown to enhance thermoelectric energy harvesting.^184^ By analogy, in-pore phase transitions in low-dimensional ionotronic systems may provide new opportunities for coupling thermal and electrochemical energy storage and conversion, although this possibility remains largely unexplored.

Beyond energy storage and conversion, first-order ionic transitions in 2D conducting confinements may enable the realization of memcapacitors—capacitors with memory^185^—through hysteretic charging behavior, with potential applications in neuromorphic systems. Earlier proposals relied on ionic transport through cylindrical nanopores in thin membranes, where the effective capacitance depended on the frequency of the applied signal.^186^ In 2D conducting confinements, charging hysteresis associated with first-order ionic transitions may provide an alternative route toward memcapacitive behavior. A key requirement, however, is the stability of metastable ionic states, and such effects have not been systematically explored.

Thus, investigating phase transitions in 2D ionotronic systems is of considerable interest, both from a fundamental scientific perspective and in view of their potential technological applications.

## 8 Conclusions and Perspectives

Low-dimensional ionotronic systems, particularly those based on 2D architectures, can unlock new opportunities across a wide range of fields. These systems enable dynamic tuning of confinement, the formation of heterostructures with pore walls composed of different materials, ballistic transport of ions and molecules, and the emergence of quantum effects—properties that are either weakly expressed or entirely absent in conventional 3D porous networks such as activated carbons, CDCs, or MOFs. We proposed a classification of low-dimensional ionotronic systems based on their electronic and confining characteristics (Section 2 and Figure 1), which can serve as a framework for systematically exploring and understanding the fundamental properties of these systems. We also reviewed the key electrode materials enabling low-dimensional ionotronics (Section 3 and Figure 2) and discussed how their unique properties may be harnessed in advanced technological applications (Section 7 and Figure 6).

Low-dimensional electrodes offer several unique advantages for electrochemical applications, particularly for rapid pseudocapacitive charge storage and delivery. The facile desorption and migration of small ions, such as protons and Li^+^, into confined regions enables ultrafast ion redistribution within the electrode. In addition, confinement can promote dense ion packing while minimizing inaccessible void space, thereby enhancing volumetric charge storage. Strategies such as pillaring 2D nanosheets or employing ions that do not cotransport solvent during charging can substantially reduce, or even eliminate, volumetric changes upon cycling. However, when low-dimensional structures are oriented parallel to the electrode surface, charging kinetics may become hindered. While vertically aligned CNT architectures exist,^187^ achieving similarly dense architectures of vertically aligned 2D nanosheets remains a significant fabrication challenge.

1D and 2D materials such as CNTs, MXenes, and graphene—with their high aspect ratios and excellent electronic conductivity—can facilitate the development of current-collector-free electrodes. Eliminating metal current collectors would not only reduce device weight, but could also broaden the range of compatible electrolytes by mitigating metal corrosion and extending the electrochemical stability window, thereby enhancing overall device performance and longevity.

An improved understanding and knowledge-driven design of low-dimensional ionotronic systems can extend their functionality far beyond conventional capacitive energy storage. Potential applications include efficient electrochemical actuation and sensing, sustainable energy harvesting, AC generation driven by oscillations in pressure, temperature, or electrolyte concentration, as well as water desalination, ion separation, and neuromorphic computing. For example, while volume changes associated with ion insertion and removal are typically detrimental to energy-storage performance, these same effects can be exploited for energy harvesting and electrochemical actuation, thereby transforming a limitation into a functional advantage. In this context, 1D and 2D solids hosting confined electrolytes offer particularly rich opportunities for controlled modulation of shape and size, enabling concepts such as soft actuators and shape-morphing structures. Moreover, the exceptional mechanical properties of materials such as MXenes open pathways toward ionotronic systems capable of withstanding pressures exceeding 1 GPa, making them attractive for high-pressure sensing applications, including load monitoring in automotive and railway technologies.

The broad range of available 2D nanomaterials—including graphene, MXenes, transition metal dichalcogenides (TMDs), and oxides—and their high degree of tunability create a vast compositional space of electrochemically active materials. By combining established physical principles with detailed atomistic modeling, artificial intelligence (AI), and machine learning (ML) approaches, this enormous electrode design space can be explored systematically, far beyond what would be feasible using simulations or experiments alone. Continued advances in AI and ML are expected to further accelerate the discovery and optimization of hybrid electrode materials, paving the way for a new generation of ionotronic systems with enhanced performance and novel functionalities.
